# Molecular Design for Optically Induced Magnetization: Targeting Excited State Orbital Degeneracy in Tungsten(V) Complexes

**DOI:** 10.1021/jacs.5c03783

**Published:** 2025-05-20

**Authors:** Ian E. Ramsier, Alysia Mandato, Sunil Saxena, Wesley J. Transue

**Affiliations:** Department of Chemistry, 6614 University of Pittsburgh, Pittsburgh, Pennsylvania 15213, United States

## Abstract

The rise of quantum information science has spurred chemists to prepare new molecules that serve as useful building blocks in quantum technologies of the future. Implementation of molecular spin-based qubits requires new methods to induce high spin polarization of samples. Herein, we report design criteria to develop axially symmetric spin-1/2 molecules amenable to optically induced magnetization (OIM), a technique using circularly polarized (CP) excitation to deliver spin polarization. We apply these criteria to develop a series of tungsten­(V) chalcogenide complexes that are demonstrated to have large spin-sensitive responses to CP light using magnetic circular dichroism (MCD) that could allow up to ∼20% spin polarization through OIM. Pulsed electron paramagnetic resonance (EPR) spectra reveal these systems have improved relaxation times over molecules like K_2_IrCl_6_, a species recently investigated by OIM, and field-swept electron spin–echo (FS-ESE) experiments show they have a remarkable lack of anisotropy in their phase-memory *T*_m_ times. The design criteria are general and point toward future ways to improve OIM-initializable qubits.

## Graphical abstract

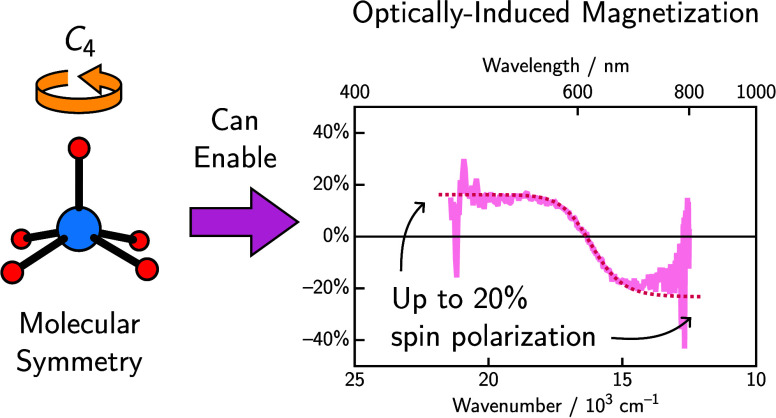

The development of molecular spin-based qubits^1^ requires that spins fulfill several criteria outlined by DiVincenzo for quantum computing^2^ or Degen for quantum sensing.^3^ Of these criteria, use of molecular spin most prominently demands advancement in methods of “initialization,” the process of selective spin polarization into a pure |0⟩ or |1⟩ state. Spin polarization is usually done thermally: application of an external magnetic field splits the *M*_S_ levels, and a Boltzmann population of these levels is then achieved on the timescale of the spin–lattice (*T*_1_) relaxation time.^4^ Conceptually, greater spin polarization can be easily reached by applying a larger magnetic field or cooling a sample further.^5^ Practically, this is difficult. The combination of low temperatures and high fields does provide high polarization, but such instrumentation^6^ can be prohibitively costly, and cooling below helium temperatures can cause increasingly slow *T*_1_ relaxation.^7,8^

Nonthermal ways to polarize spin sidestep these issues, and optical methods have proven especially promising.^9–11^ These techniques leverage electronic excitation and molecular design to influence spin, allowing chemists to untether spin polarization from Boltzmann population and spin *T*_1_ relaxation. We are interested in a spin-sensitive optical phenomenon known as “optically induced magnetization” (OIM). OIM is deeply intertwined with a technique more familiar to chemists, magnetic circular dichroism (MCD): MCD measures the differential response of *M*_S_ levels to circularly polarized (CP) light (Δϵ = ϵ_LCP_ – ϵ_RCP_),^12,13^ whereas OIM exploits this differential response to preferentially bleach one *M*_S_ level. Despite demonstration of OIM in a wide variety of systems,^14–18^ the method has not been well-explored to manipulate molecular spin. This will surely change after a recent report demonstrating the usefulness of OIM to explore ultrafast spin dynamics of K_2_IrCl_6_.^19^

Selection of K_2_IrCl_6_ relied on its octahedral geometry and its ^2^ *T*_2*g*_ ground state (GS) orbital angular momentum (OAM), promoting a large MCD/OIM response but also causing exceedingly short coherence (phase-memory) *T*_m_ times, even at 20 K.^19^ Such rapid decoherence impairs many applications in quantum technologies by limiting the computations that can be performed or the information that may be sensed by a qubit.^2,3^ Herein, we report a more flexible molecular design strategy to optimize OIM responses that allows us to explore the preparation of *S* = 1/2 molecules of axial symmetry that lack any GS OAM. Our design strategy led us to a series of tungsten­(V) chalcogenide complexes [**1**·E]^−^ (Figure 1a) that could give up to ∼20% spin polarization by OIM. Pulsed electron paramagnetic resonance (EPR) experiments reveal improved spin relaxation characteristics for these complexes due to their lack of GS OAM or a low-lying excited state (ES), and field-swept electron spin–echo (FS-ESE)^21^ EPR measurements reveal a remarkable insensitivity of phase-memory (*T*_m_) times to magnetic field.

**Figure 1:**
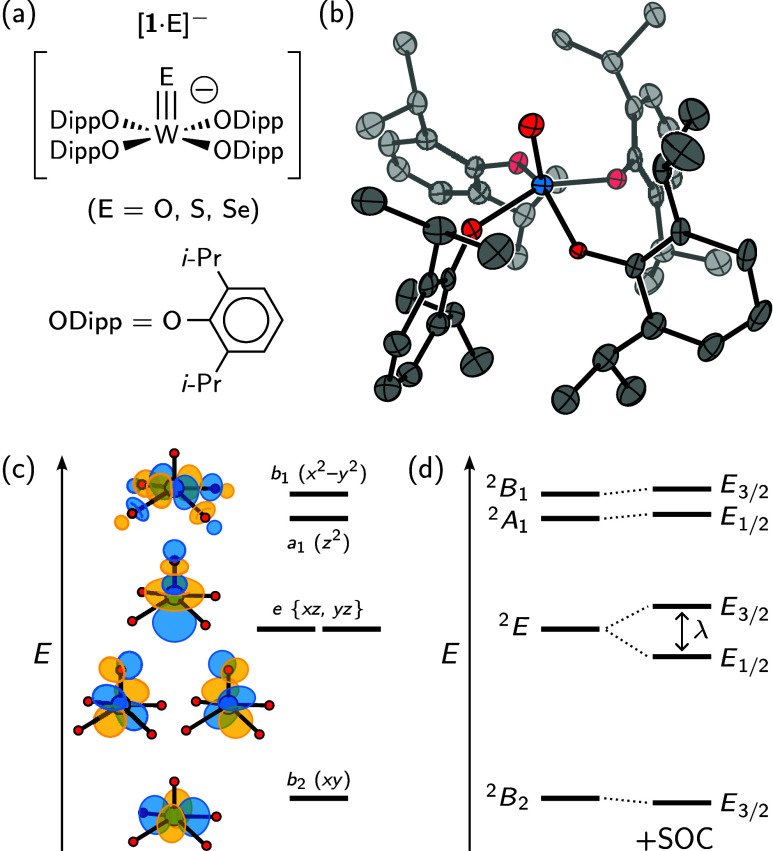
(a) The [**1**·E]^−^ compounds have bulky aryloxide ligands that enforce pseudo-*C*_4*v*_ geometry, confirmed by (b) the crystal structure of [Na­(THF)_6_]­[**1**·O]. (c) The *d* orbital splittings for [**1**·O]^−^ are shown with *C*_4*v*_ irrep labels. (d) SOC splits the ^2^ *E* state into two components labeled by *C*_4v_^*^ double group irreps.^20^

Using the language of MCD theory, maximization of OIM is equivalent to maximization of the “*C* term” MCD response, something driven by spin–orbit coupling (SOC) in transition-metal systems.^22^ The K_2_IrCl_6_ system experienced strong SOC due to the residual (“fictitious”)^4^ OAM in its GS, but there is no reason SOC splitting must come from the GS. Instead, we were inspired by ruby, where OIM can be performed from its orbitally nondegenerate ^4^ *A*_2_ GS to a degenerate ^2^ *E* ES.^14^ This ^2^ *E* ES of ruby lacks residual OAM; yet, SOC still causes splitting into two Kramers doublets. Because SOC is the dominant driver of this splitting, spin and orbital angular momenta strongly mix to deliver a highly *M*_S_-selective MCD/OIM response.^23^ Clearly, threefold orbital degeneracy is not required for OIM.

Simply put, any point group possessing degenerate irreducible representations (irreps) may allow for orbital-based optimization of OIM. This certainly includes cubic point groups (*O*_*h*_, *T*_*d*_, etc.), but also all axial point groups of threefold-or-higher symmetry. At its simplest, OIM maximization should therefore incorporate

- axial (*C*_(*n* ≥ 3)(*v*/*h*)_, *D*_(*n* ≥ 3)(*h*)_, *D*_(*n* ≥ 2)*d*_, *S*_2*n* ≥ 4_) or isometric (*T*_(*d*/*h*)_, *O*_(*h*)_, *I*_(*h*)_) point groups
- an optical transition involving an orbitally degenerate state
- SOC splitting of similar or greater magnitude to the line width of the electronic transition.

^18^ An OIM-initializable qubit should additionally target increased spin coherence through

- avoidance of GS OAM and low-lying ESs
- a large absorptivity (ϵ) to allow more dilute samples

Of the few examples of molecular OIM we are aware of,^18,19^ every system fails to satisfy at least one of these five criteria.

We selected tungsten­(V)–oxo complex [**1**·O]^−^ as a promising system: tetragonal *d* ^1^ oxos generally have a low-lying ^2^ *E* ES (Figure 1c),^24^ and 5*d* metals have strong SOC (ζ_W(V)_ = 3483 cm^–1^).^25^ Heating a THF solution of WOCl_3_(THF)_2_ ^26^ and sodium 2,6-diisopropylphenoxide (NaODipp, 4 equiv) overnight (69 °C, 18 h) provided deep blue [Na­(THF)_6_]­[**1**·O] in 50% recrystallized yield. A preliminary X-ray diffraction study (Figure 1b) revealed a nearly *C*_4_-symmetric anion with a geometry close to an ideal square pyramid (τ_5_ = 0.052(6)).^27^ An optical ^2^ *B*_2_ → ^2^ *E* transition was observed in the UV–vis–NIR absorption spectrum (16 100 cm^–1^, Figure 2a) with a prominent shoulder due to tungsten­(V) SOC, and a relatively large absorptivity for a *d*–*d* transition (270 M^–1^ cm^–1^). Thus, [**1**·O]^−^ satisfied all our design requirements.

**Figure 2:**
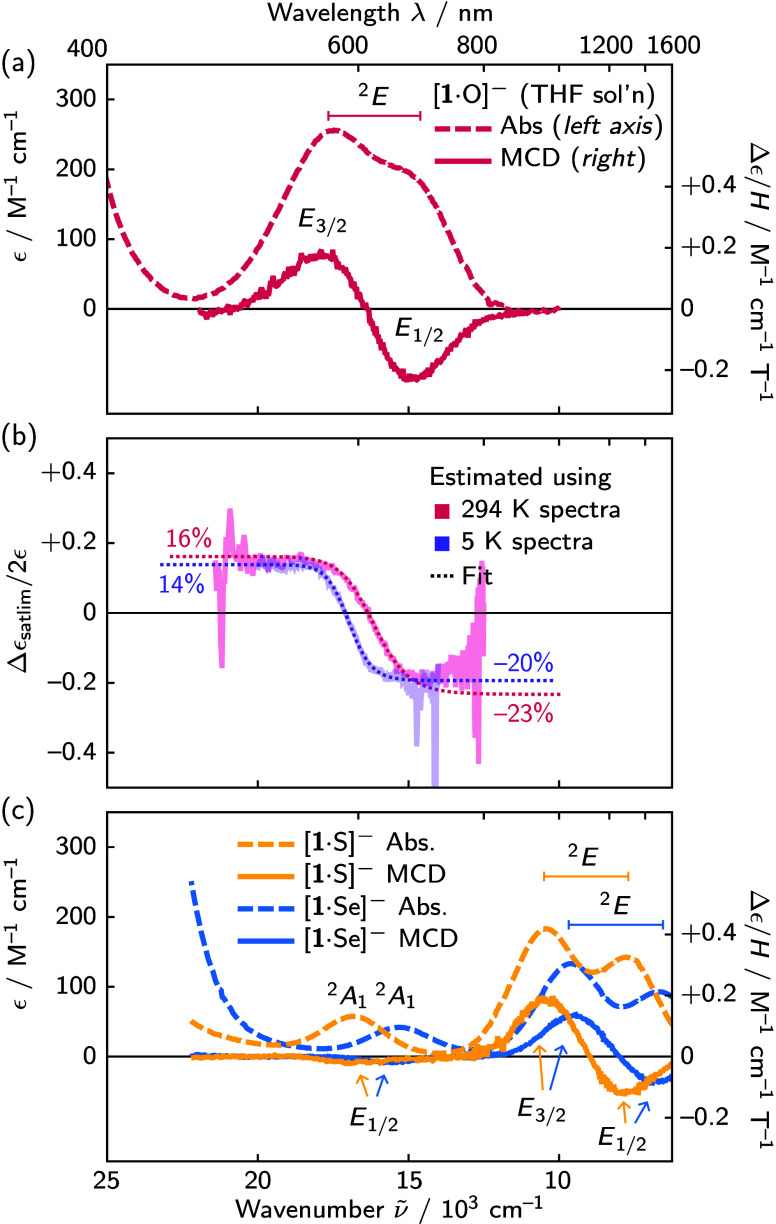
(a) Room-temperature UV–vis–NIR absorption and MCD spectra of [Na­(THF)_6_]­[**1**·O] (THF solution). (b) The maximum spin polarization through OIM is |Δϵ_satlim_/2ϵ|, estimated here for [**1**·O]^−^ in two ways (see text and the SI). (c) The absorption and MCD spectra of the heavier chalcogenide complexes reveal a ^2^ *A*_1_ transition.

We were pleased to find the MCD spectrum of [**1**·O]^−^ shows large intensities associated with its ^2^ *E* ES. The SOC splitting of this state was clearly indicated by the characteristic bisignate (“pseudo-*A*”) line shape, the negative and positive features corresponding to *E*_1/2_ and *E*_3/2_ levels, respectively (see the SI). The MCD intensities of these levels were quantified through their “*C*_0_/*D*_0_” ratios, a ratio of MCD *C* term intensity (“*C*_0_”) to absorption intensity (“*D*_0_”). For a simple spin-1/2 GS lacking OAM and low-lying ESs, the maximum possible magnitude of |*C*_0_/*D*_0_| is *g*/2, half the GS *g* value (see the derivation given in the SI); however, molecules typically feature ratios far smaller (Chart 1). The values for the [**1**·O]^−^^2^ *B*_2_  → ^2^ *E* transitions (−0.46(3) and +0.32(4)) were quite large, multiple times larger than those of TEMPO^•^ and Cu­(acac)_2_ exemplars, and approach the values for ${\left[{IrCl}_{6}\right]}^{\mathrm{2-}}$ ,^13,28^ an ion specifically chosen for OIM studies due to its residual GS OAM.^19^

**Chart 1:**
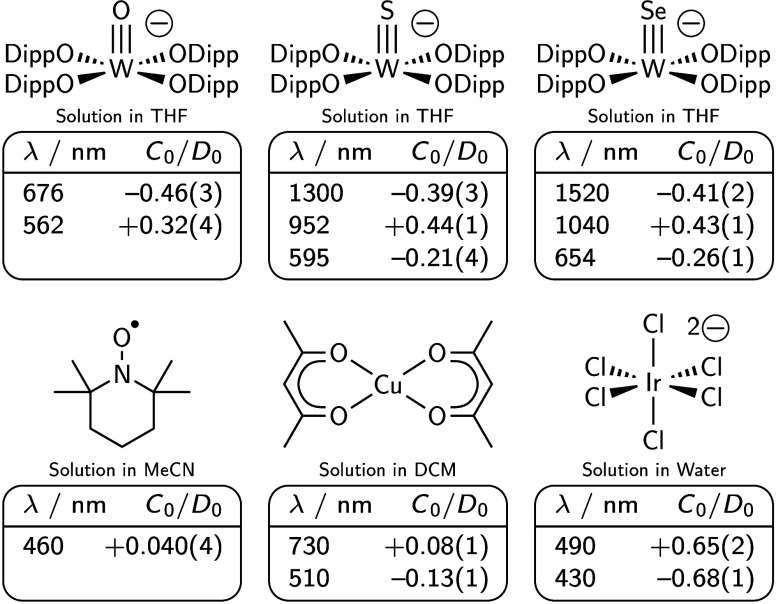
^2^ *B*_2_ → ^2^ *E* Transitions of [**1**·E]^−^ Species Have Far Stronger MCD Intensities than Other *S* = 1/2 Organic or Transition Metal Species with Smaller SOC; Their *C*_0_/*D*_0_ Ratios Approach Those of **[IrCl_6_]^2–^**, a System with GS OAM

The MCD *C*_0_/*D*_0_ ratio is a useful heuristic, but more direct insight for OIM would come from measuring Δϵ for each individual *M*_S_ level. This can be accomplished in the saturation limit. Magnetic saturation occurs under sufficiently low temperatures or high fields that most molecules populate a single *M*_S_ level. In the limit of complete saturation, the MCD spectrum reveals the intrinsic Δϵ for the single lowest-energy *M*_S_ level. MCD intensity in the presence of saturation is nonlinear,^29^

1 $$\frac{\Delta \epsilon }{E}=\gamma \frac{2C_{0}}{g}\;\tanh\left(\frac{g\mu_{B}B}{2k_{B}T}\right)f\left(E\right)$$

where *E* is the excitation energy, γ a proportionality constant, μ_B_ the Bohr magneton, *B* the magnetic field, *k*_B_ the Boltzmann constant, *T* the temperature, and *f*(*E*) a line shape function (see the SI). We used eq 1 to estimate MCD in the saturation limit Δϵ_satlim_ in two ways: from room-temperature data, and from cryogenic MCD data (5 K, 7 T). The maximum spin polarization that can be achieved by OIM is |Δϵ_satlim_/2ϵ| (see the SI), and both methods of estimating Δϵ_satlim_/2ϵ reveal that CP excitation of the ^2^ *B*_2_ → ^2^ *E* transition can deliver up to 15% (*E*_3/2_) or 20% (*E*_1/2_) spin polarization, depending on wavelength of excitation (Figure 2b). For context, such a large polarization can only be achieved thermally at 1–1.5 K in X-band EPR experiments.

Analogous chalcogen congeners [**1**·S]^−^ and [**1**·Se]^−^ were also synthesized to explore their MCD intensities. The former was prepared by treating W­(ODipp)_4_ ^30^ with NaSCPh_3_ (1 equiv, THF), a convenient source of S^• –^ upon release of CPh_3_^•^. Stirring overnight then washing with ether provided teal [Na­(THF)_6_]­[**1**·S] in 36% yield after recrystallization. The latter complex was prepared in a two-step one-pot procedure: W­(ODipp)_4_ was first treated with PPh_3_Se to form the terminal selenide complex, and then reduction with NaCPh_3_ formed the tungsten­(V) anion. Recrystallization provided vibrant green [Na­(THF)_*x*_(Et_2_O)_*y*_]­[**1**·Se] in 26% isolated yield.

The MCD spectra of [**1**·S/Se]^−^ show the appearance of the ^2^ *B*_2_ → ^2^ *A*_1_ ligand field transition, likely caused by weaker σ_W≡E_ interactions in the heavier chalcogenides. This assignment follows from its negative MCD response, indicating *E*_1/2_ symmetry, and is corroborated by model CASSCF/RI-NEVPT2 calculations (see the SI).^31^ We ascribe the weaker |*C*_0_/*D*_0_| ratios (Chart 1) of the ^2^ *B*_2_ → ^2^ *A*_1_ transitions to a lack of ES orbital degeneracy. For such nondegenerate states, the sum-over-states perturbation theory formalism^22^ says *C* term intensity is proportional to the reciprocal energy difference to other states coupling through SOC. This is an important distinction from ^2^ *E* states, underscoring that design of molecules with large MCD/OIM responses should target transitions involving orbital degeneracy.

All three chalcogenide complexes can be observed by continuous-wave (CW) EPR experiments at temperatures at least up to 80 K due to their lack of GS OAM and thus slower relaxation properties. Inversion recovery and Hahn-echo X-band EPR experiments were used to directly measure *T*_1_ and *T*_m_ times (Figure 3), giving values higher than those of K_2_IrCl_6_ (10–100 times higher at 20 K);^19^ in fact, even higher values may be attainable through techniques like deuteration,^32^ picket-fence saturation recovery experiments,^33^ and Carr–Purcell–Meiboom–Gill (CPMG) pulse sequences.^34^ Despite this increase in coherence for [**1**·E]^−^ complexes, these *T*_*m*_ relaxation times are still modest likely due to the strong SOC within the systems.^35^ A balance clearly needs to be struck between maximizing MCD/OIM using heavy atom SOC while avoiding the collapse of spin coherence, and this will be a fascinating area for future exploration.

**Figure 3:**
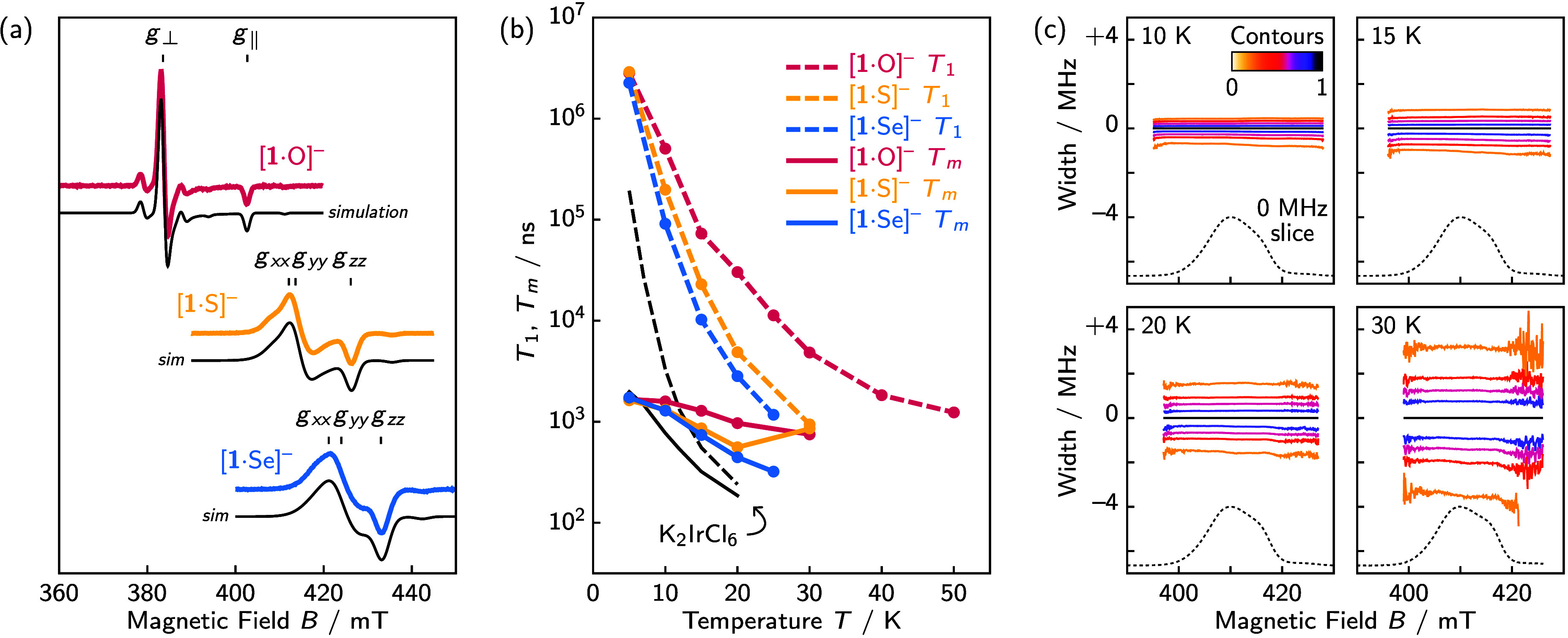
(a) The CW EPR spectra show approximate *C*_4_ symmetry for all anions with slight rhombic distortions for [**1**·S/Se]^−^. (b) Spin *T*_1_ and *T*_m_ measurements show slower relaxation for [**1**·E]^−^ anions than K_2_IrCl_6_ (values from ref 19) due to the latter’s GS OAM. (c) Normalized contours in the FS-ESE spectrum of [**1**·Se]^−^ show nearly flat *T*_m_ times independent of field.

The *T*_1_ and *T*_m_ measurements also showed unexpectedly consistent relaxation times when measured in the *g*_∥_ and *g*_⊥_ regions of the spectra. Such behavior differs from related species,^36^ which often show large variations across the spectra.^37^ This insensitivity to magnetic field was demonstrated using a 2D FS-ESE EPR experiment^21^ conducted on [**1**·Se]^−^, showing nearly constant *T*_m_ times across the entire spectrum. Studies are ongoing to uncover correlations between relaxation times, molecular symmetry, and MCD/OIM intensity.

With this work, we hope to highlight that a large synthetic space remains to be explored in the development of optimal molecules for OIM. Our results ease the restrictive *O*_*h*_ design strategy for OIM, showing that chemists can exploit axial symmetry to deliver large MCD responses. This increased flexibility should allow targeting of other properties important for qubits, especially spin relaxation times. Ligand design may be particularly useful, as the ODipp ligands here were selected for ease of use^30^ rather than promotion of spin coherence.^38–40^ We are conducting experiments toward these ends, and toward balancing spin and optical lifetimes for future implementation of OIM in molecular qubit systems on the nanosecond time scale.^41^

## Supplementary Material



## Notes

Experimental details, spectroscopic characterization, crystallographic information, computational details, and theoretical derivations (PDF)
