# Supplementary material for: Molecular Design for Optically Induced Magnetization: Targeting Excited State Orbital Degeneracy in Tungsten(V) Complexes
Source: J Am Chem Soc. 2025 May 20;147(22):18424–30. doi: 10.1021/jacs.5c03783 (PMC12147145; doi:10.1021/jacs.5c03783)
Supplement: Supplementary file 1 [file ja5c03783_si_001.pdf]

Molecular Design for Optically Induced Magnetization: Targeting Excited State Orbital Degeneracy in Tungsten(V) Complexes

Ian E. Ramsier, Alysia Mandato, Sunil Saxena, Wesley J. Transue

Supporting Information

Contents

S1 Synthesis **2**
S1.1 Synthesis of Sodium Tritylthiolate ($NaSCPh_3$) 2
S1.2 Synthesis of Tritylsodium ($NaCPh_3$) 2
S1.3 Synthesis of Sodium 2,6-Diisopropylphenolate (NaODipp) 3
S1.4 Synthesis of $[Na(THF)_6][WO(ODipp)_4]$ ([**1**·O]) 3
S1.5 Synthesis of $[Na(THF)_6][WS(ODipp)_4]$ ([**1**·S]) 3
S1.6 Synthesis of $[Na(THF)_x(Et_2O)_y][WSe(ODipp)_4]$ ([**1**·Se]) 4
S1.7 Initial Attempts to Prepare $[Na(THF)_x][WTe(ODipp)_4]$ ([**1**·Te]) 4

S2 MCD Spectroscopy **5**
S2.1 Data Acquisition and Processing 5
S2.2 Fitting C_0/D_0 Ratios from Room Temperature Spectra 5
S2.2.1 $[Na(THF)_6]$[**1**·O] 7
S2.2.2 $[Na(THF)_6]$[**1**·S] 9
S2.2.3 $[Na(THF)_x(Et_2O)_y]$[**1**·Se] 11
S2.2.4 TEMPO 13
S2.2.5 Potassium Hexachloroiridate(IV) 15
S2.2.6 Copper(II) Acetylacetonate 17
S2.3 Cryogenic MCD Studies of $[Na(THF)_6]$[**1**·O] 19
S2.4 Theoretical Considerations 20
S2.4.1 Predicting Signs of C Term Intensity Using the Wigner–Eckart Theorem 20
S2.4.2 MCD Saturation Behavior and Maximal $|C_0/D_0|$ Ratios 22
S2.4.3 Maximum Spin Polarization through OIM and Figure 2(b) 23

S3 Electron Paramagnetic Resonance **24**
S3.1 Continuous Wave EPR Experiments 24
S3.2 Hahn Spin-Echo EPR Experiments 25
S3.3 Two-Dimensional Field-Swept Electron Spin-Echo Experiments 25

S4 X-ray Crystallography **28**

S5 Computational Methods **33**
S5.1 CASSCF/RI-NEVPT2 Multireference Calculations 33
S5.1.1 A Minimal (1,5) Active Space 33
S5.1.2 Larger (5,7) and (9,9) Active Spaces and the Role of Covalency 34
S5.2 Optimized $[WE(ODipp)_4]^-$ XYZ Coordinates 38
S5.3 Model Complex $[WE(OPh)_4]^-$ XYZ Coordinates 45

S1 Synthesis

All manipulations were performed in a MBraun LabMaster Pro SP glove box under an inert atmosphere of purified N_2 or using standard air-free Schlenk techniques. All solvents were obtained anhydrous and oxygen-free by bubble degassing (Ar), purification through alumina columns (Pure Process Technology Solvent Purification System), and storage in the glove box over 3 Å activated molecular sieves. Deuterated solvents were degassed and stored over molecular sieves for at least 2 days prior to use. Glassware was oven-dried for at least 2 h at temperatures greater than 150 °C.

Sodium hexamethyldisilazide (solid, Thermo Scientific), triphenylmethanethiol (Thermo Scientific), sodium *tert*-butoxide (Thermo Scientific), *n*-butyllithium (2.5 M in hexanes, Strem), triphenylmethane (Thermo Scientific), and triphenylphosphine selenide (Thermo Scientific) were purchased and used as received. Literature compounds $WOCl_3(THF)_2$,[1] $W(ODipp)_4$,[2,3] and tricyclohexylphosphine telluride (PCy_3Te)[4] were prepared according to the reported procedures.

All NMR spectra on diamagnetic compounds were obtained on Bruker Avance III (400 or 500 MHz) instruments. ^{1}H and ^{13}C NMR chemical shifts (δ) are reported in parts per million (ppm) relative to tetramethylsilane, using residual proton signals from the deuterated solvent as an internal reference.[5] High resolution mass spectrometry (HRMS) were obtained on a Thermo Scientific Q-Exactive mass spectrometer through electrospray ionization (ESI); the mobile phase was acetonitrile spiked with formic acid.

S1.1 Synthesis of Sodium Tritylthiolate ($NaSCPh_3$)

$$\mathrm{HSCPh_3} \xrightarrow[\mathrm{Et_2O,\ 1\ h}]{\mathrm{NaN(SiMe_3)_2} \atop -\ \mathrm{HN(SiMe_3)_2}} \mathrm{NaSCPh_3}$$

A solution of $NaN(SiMe_3)_2$ (371 mg, 1.55 mmol, 1.07 equiv) in diethyl ether (4 mL) was added to a rapidly stirring solution of trityl mercaptan (523 mg, 1.45 mmol, 1 equiv) in diethyl ether (11 mL). After stirring 1 h, the solution was concentrated under vacuum until a white solid precipitated. Collecting the white precipitate on a medium porosity frit and drying under reduced pressure yielded 409 mg (1.1 mmol, 75%). ^{1}H NMR (400 MHz, DMSO-d_6) δ 7.45 (d, J = 7.8, 6H, *o*-CH), 7.00 (t, J = 7.5, 6H, *m*-CH), 6.90 (t, J = 7.2, 3H, *p*-CH). ^{13}C NMR (100 MHz, DMSO-d_6) δ 155.8 (*ipso*-C), 129.8 (*o*-C), 125.5 (*m*-C), 123.0 (*p*-C), 62.6 (C–S).

S1.2 Synthesis of Tritylsodium ($NaCPh_3$)

$$\mathrm{HCPh_3} \xrightarrow[\mathrm{hexanes} \atop \mathrm{overnight}]{t\text{-}\mathrm{BuONa},\ n\text{-}\mathrm{BuLi} \atop -\ t\text{-}\mathrm{BuOLi},\ n\text{-}\mathrm{BuH}} \mathrm{NaCPh_3}$$

$NaCPh_3$ was prepared following a modified literature procedure.[6] Sodium *tert*-butoxide (120 mg, 1.25 mmol, 1.0 equiv) was weighed into a vial and suspended in hexanes, then *n*-butyllithium (0.5 mL, 1.25 mmol, 2.5 M in hexanes, 1.0 equiv) was added by syringe. The opaque white suspension was stirred for ∼30 min, then it was added to a suspension of triphenylmethane (305 mg, 1.25 mmol, 1.0 equiv) in hexanes. The color gradually changed to orange and then blood red as the mixture stirred overnight, and a red precipitate developed. The precipitate was collected on a fine porosity frit and washed with hexanes (3 × 4 mL); these red-orange hexane washes were discarded. Crude product was dried in the frit under vacuum, yielding 157 mg of a fine red powder. Recrystallization by cooling a concentrated ether solution to −35 °C gave dark red crystals (90 mg, 0.34 mmol, 28%), which were dried and used without further purification. The material is sensitive, but we have found it can be stored cold (−35 °C) within the glovebox freezer for 1–2 weeks without noticeable impact on our chemistry.

S1.3 Synthesis of Sodium 2,6-Diisopropylphenolate (NaODipp)

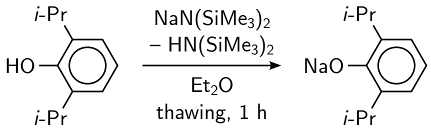

A yellow solution of 2,6-diisopropylphenol HODipp (1.00 g, 5.60 mmol) was prepared with diethyl ether (4 mL) and was frozen in the glovebox cold well. A separate vial was charged with $NaN(SiMe_3)_2$ (1.08 g, 5.88 mmol) and diethyl ether (10 mL) which formed a light yellow solution which was also frozen in the cold well. Upon thawing, the $NaN(SiMe_3)_2$ solution was added dropwise to the stirring light yellow HODipp solution, causing a white precipitate to develop. After stirring 30 min, the white solids were collected, washed with diethyl ether, and dried under reduced pressure to a fine white powder (1.09 g, 79.7%) that was used without further purification. ^{1}H NMR (400 MHz, DMSO-d_6) δ 6.53 (d, J = 7.3, 2H, m-CH), 5.79 (t, J = 7.22, 1H, p-CH), 3.37 (m, J = 7.0, 2H, CH), 1.02 (d, J = 6.95, 12H, CH_3). ^{13}C NMR (100 MHz, DMSO-d_6) δ 167.2 (C-O), 134.7 (o-C), 121.6 (m-C), 105.5 (p-C), 26.2 (CH), 24.1 (CH_3).

S1.4 Synthesis of [Na(THF)$_6$][WO(ODipp)$_4$] ([1·O])

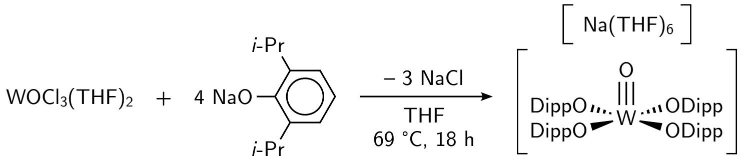

A colorless solution of NaODipp (133 mg, 0.544 mmol, 4 equiv) in THF (3 mL) was prepared and frozen in the glovebox cold well. Separately, a teal suspension of $WOCl_3(THF)_2$ (61 mg, 0.136 mmol, 1 equiv) in THF (4 mL) was prepared and frozen. Upon thawing, the NaODipp solution was added dropwise to the stirring teal mixture. During the course of addition, the mixture became a dark blue, then a green, and finally a dark brown solution. The reaction mixture was allowed to stir and warm to room temperature for 30 min. The brown solution was transferred into a heavy-walled glass pressure vessel and moved to a pre-heated oil bath (69°C) and stirred overnight (18 hrs), during which time it turned dark blue and off-white precipitate had formed. After cooling, the flask was returned to the glovebox. White solids (NaCl) were removed by filtration through microfiber glass filter paper and the dark blue filtrate was dried under reduced pressure. The resulting solids were recrystallized via THF/ether vapor diffusion (−35 °C), yielding 88 mg (0.068 mmol, 50%) dark blue crystals. HRMS (ESI, MeCN) m/z: $[M + CO_2H]^-$ Calc'd for $C_{49}H_{69}O_7W$ 953.4547; Found 953.4548.

S1.5 Synthesis of [Na(THF)$_6$][WS(ODipp)$_4$] ([1·S])

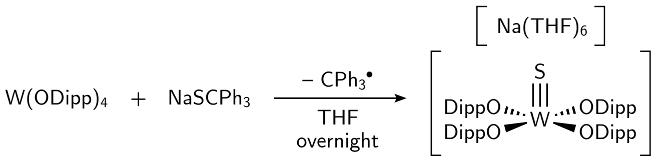

A suspension of $NaSCPh_3$ (33.3 mg, 0.112 mmol, 1 equiv) in THF (2 mL) was added to a dark green solution of $W(ODipp)_4$ (100 mg, 0.112 mmol, 1 equiv) in THF (4 mL) with rapid stirring. The solution rapidly became homogeneous, and continued vigorous stirring for 24 h resulted in a gradual color change from dark green to amber–yellow. Stripping volatiles under reduced pressure gave a brown residue, and washing with diethyl ether (3×3 mL) left a teal colored powder weighing 82 mg. Recrystallization by vapor diffusion of diethyl ether into a concentrated THF solution at −35 °C yielded 54 mg (0.040 mmol, 36%) teal crystals. This material was notably more sensitive than [Na(THF)$_6$][**1**·O] and was found to gradually decompose upon storage; the compound was always prepared and then used immediately after recrystallization. HRMS (ESI, MeCN) m/z: $[M + CO_2H]^-$ Calc'd for $C_{49}H_{69}O_6SW$ 969.43187; Found 969.43286.

S1.6 Synthesis of [Na(THF)$_x$(Et$_2$O)$_y$][WSe(ODipp)$_4$] ([1·Se])

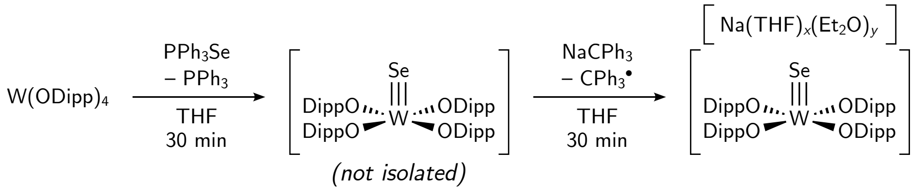

A colorless solution of PPh_3Se (38.2 mg, 0.112 mmol, 1.0 equiv) in minimal THF (∼2 mL) was rapidly added to a stirring dark green solution of W(ODipp)$_4$ (100 mg, 0.112 mmol, 1.0 equiv) in THF (4 mL). The color quickly changed from green to a vibrant red, presumably indicating formation of the neutral WSe(ODipp)$_4$ species. After stirring 30 min, a vibrant red solution of $NaCPh_3$ (29.8 mg, 0.112 mmol, 1.0 equiv) in minimal THF (∼2 mL) was added to the reaction mixture. During addition, the color changed to yellow and then back to deep red. The reaction mixture was stirred 30 min and then dried completely under vacuum to give a red residue. Washing with diethyl ether (3 × 4 mL) removed the red color to reveal a bright green powder that was dried under vacuum. Minimal THF (∼1 mL) was used to dissolve this crude green material (∼70 mg), and product was recrystallized by vapor diffusion of ether at −35 °C over several days, yielding 42 mg (0.03 mmol, 26%) vibrant green crystals. This material was the most sensitive of the trio, and was found to gradually decompose even when stored in the glovebox freezer; the compound was always prepared and then used immediately after recrystallization. HRMS (ESI, MeCN) m/z: [M + CO_2H]$^-$ Calc'd for $C_{49}H_{69}O_6SeW$ 1017.37632; Found 1017.37866.

We have found the solvation of the sodium cation to be variable. We have obtained crystal structures with a [Na(THF)$_{4.5}$(Et$_2$O)$_{0.5}$]$^+$ cation composition and a [Na(THF)$_6$]$^+$ cation composition (due to poor data quality of the former, only this latter structure is included herein). Upon extended drying under vacuum, the crystals visibly crumble into a powder. We have attempted to quantify the sodium solvation shell after extended drying by dissolution in acetonitrile-d_3 and then storage outside the glovebox overnight to allow for gradual autoxidation into diamagnetic products. Integration has provided variable compositions with roughly four ethereal solvents per sodium. When preparing samples for spectroscopic analysis, we have assumed a composition of [Na(THF)$_{4.5}$(Et$_2$O)$_{0.5}$]$^+$, which gives a rough composition that we believe to be of sufficient accuracy for our MCD C_0/D_0 investigations.

S1.7 Initial Attempts to Prepare [Na(THF)$_x$][WTe(ODipp)$_4$] ([1·Te])

A yellow solution of PCy_3Te (20 mg, 0.048 mmol, 1.0 equiv) in minimal THF was added to a rapidly stirring dark green solution of W(ODipp)$_4$ (43 mg, 0.048 mmol, 1.0 equiv). The color quickly changed to maroon, presumably indicating formation of WTe(ODipp)$_4$. After stirring 30 min, a vibrant red solution of $NaCPh_3$ was slowly added to the reaction mixture. No changes in color were obvious; the mixture remained red-brown. After stirring 30 min, removal of volatiles under vacuum provided a brown residue that was soluble in diethyl ether. Attempts to recrystallize from diethyl ether in the glovebox freezer (−35 °C) did not produce crystals. This is not to say we think the [**1**·Te]$^-$ anion cannot be prepared; we only wish to provide documentation of some initial attempts that were unsuccessful.

S2 MCD Spectroscopy

S2.1 Data Acquisition and Processing

Absorption and magnetic circular dichroism (MCD) spectroscopies were performed using a JASCO J-1700 spectropolarimeter with equipped with a S-20 photocathode-equipped photomultiplier tube (PMT) detector ($\lambda = 163-950$ nm, JASCO Model PM-539), a S-1 photocathode-equipped PMT detector ($\lambda = 400-1250$ nm, JASCO Model EXPM-531), or an InGaAs detector ($\lambda = 800-1600$ nm JASCO Model EXIG-542 or $\lambda = 1600-2500$ nm EXIG-543). Room-temperature MCD data were acquired using a JASCO MCD-581 electromagnet ($|B| \leq 1.5$ T); samples held within the electromagnet setup were generally found to be 21 ± 1 °C, so a temperature of 294 K has been used throughout room temperature data processing. Cuvettes were purchased from Spectrocell (1 or 10 mm "NIR" quartz cells, 220–3500 nm range), and some were adapted to have a Young's valve to allow for analysis of air-free samples.

Cryogenic MCD data were acquired using an Oxford SpectromagPT magnet ($T = 1.7-300$ K, $|B| \leq 7$ T). Samples were contained between two quartz windows (Spectrocell NIR quartz, 1 cm diameter) and a fluoropolymer O-ring, then flash-frozen in a liquid nitrogen bath to obtain a glass, and loaded into the magnet cold. The JASCO J-1700 spectropolarimeter was arranged with its spectrometer and detector located on opposite sides of the magnet, ensuring the detector was outside the 50 G line, and three planoconvex CaF_2 lenses were used to focus light through the sample. Depolarization was checked using a nickel(II) tartrate solution before/after the sample compartment to ensure less than 5% loss in CD intensity.

Data were acquired at a 1 nm data pitch and other relevant parameters (scanning speed, detector integration time, number of accumulations, and bandwidth) were chosen to maximize the signal-to-noise ratio while preventing distortion of features. As discussed in the J-1700 manual, the product of the detector integration time and the scanning speed limits the resolution of peaks that may be discerned and should be chosen to be less than 1/10 the peak width; we generally used 200 nm/min scanning rates and a 1024 ms detector integration time (product: 3.4 nm) unless peaks appeared to be narrower than 30 nm. When multiple detectors were necessary to fully characterize the room temperature MCD spectra of a compound, the same sample was used across all regions. Solvent blanks were collected under identical conditions to the absorption/MCD spectra of the samples, and were subtracted from the final data.

Room temperature spectra were acquired at a series of field strengths (e.g. 0, ±1, ±1.5 T). These experimental conditions are in the linear limit ($\mu_B B/k_B T \ll 1$), giving MCD intensity strictly proportional to the applied field. Under these linear conditions, the field-independent and field-dependent components of the measured ellipticity data were separated using a Moore–Penrose pseudoinverse. For example, collection of five spectra over the 300–800 nm region at $B = 0, \pm 1, \pm 1.5$ T field strengths allows extraction of the baseline (field-independent $I^{(0)}$) and MCD (field-dependent $I^{(1)}$) components through

$$\begin{pmatrix} 1 & 1.5 \\ 1 & 1 \\ 1 & 0 \\ 1 & -1 \\ 1 & -1.5 \end{pmatrix}^{\ominus} \begin{pmatrix} I^{+1.5\text{ T}}_{300\text{ nm}} & I^{+1.5\text{ T}}_{301\text{ nm}} & I^{+1.5\text{ T}}_{302\text{ nm}} & \cdots & I^{+1.5\text{ T}}_{800\text{ nm}} \\ I^{+1.0\text{ T}}_{300\text{ nm}} & I^{+1.0\text{ T}}_{301\text{ nm}} & I^{+1.0\text{ T}}_{302\text{ nm}} & \cdots & I^{+1.0\text{ T}}_{800\text{ nm}} \\ I^{0.0\text{ T}}_{300\text{ nm}} & I^{0.0\text{ T}}_{301\text{ nm}} & I^{0.0\text{ T}}_{302\text{ nm}} & \cdots & I^{0.0\text{ T}}_{800\text{ nm}} \\ I^{-1.0\text{ T}}_{300\text{ nm}} & I^{-1.0\text{ T}}_{301\text{ nm}} & I^{-1.0\text{ T}}_{302\text{ nm}} & \cdots & I^{-1.0\text{ T}}_{800\text{ nm}} \\ I^{-1.5\text{ T}}_{300\text{ nm}} & I^{-1.5\text{ T}}_{301\text{ nm}} & I^{-1.5\text{ T}}_{302\text{ nm}} & \cdots & I^{-1.5\text{ T}}_{800\text{ nm}} \end{pmatrix} = \begin{pmatrix} I^{(0)}_{300\text{ nm}} & I^{(0)}_{301\text{ nm}} & I^{(0)}_{302\text{ nm}} & \cdots & I^{(0)}_{800\text{ nm}} \\ I^{(1)}_{300\text{ nm}} & I^{(1)}_{301\text{ nm}} & I^{(1)}_{302\text{ nm}} & \cdots & I^{(1)}_{800\text{ nm}} \end{pmatrix},$$

where "$\ominus$" indicates the pseudoinverse. The first column of ones in the leftmost matrix indicates that the baseline is constant among the data sets, and the second column of the leftmost matrix contains the strengths of the applied fields. The output gives the field-independent ellipticity in its first row (i.e. the baseline), and the field-dependent ellipticity in its second row (i.e. MCD expressed in millidegrees per tesla). The field-dependent ellipticity $[\theta]$ was converted to $\Delta\epsilon$ MCD intensity by $\Delta\epsilon = [\theta\,(\text{mdeg})]/(32982Cl)$ using concentration C and path length l.

S2.2 Fitting C_0/D_0 Ratios from Room Temperature Spectra

The fitting of C_0/D_0 ratios is challenging due to the difficulties introduced by lineshape, baseline, scattering, and overlapping transitions, and this is particularly true for the absorption data.[7] Better fits can come through simultaneously fitting both absorption and MCD data, allowing shared wavenumbers $\tilde{\nu}$ and peak widths σ between the data sets to increase the certainty in each fitted parameter.

When approaching simultaneous fits of multiple data sets, a question inherently arises: how does one weigh each data set in the fit? If one attempts to fit raw absorption A versus MCD ΔA from room temperature data, the MCD data will be roughly 10^{3-4} smaller than the absorption data. This difference in magnitude gives a de-facto 10^{3-4}:1 weighting scheme, and causes wavenumbers and peak widths to be fit almost exclusively to the absorption data, undermining the utility of simultaneous fitting. Instead, we have chosen to simultaneously fit absorption A and $(\Delta A)(k_BT/\mu_BB) \approx \Delta A_{\text{satlim}}$ for room temperature data sets, scaling the MCD intensity to the saturation limit. We felt this selection allowed us to standardize data processing between compounds, to minimize arbitrariness in the weighing scheme, and to allow simultaneous fitting that is less biased towards the absorption data. After scaling MCD to the estimated saturation limit, the following equations were used to fit the spectra: (note: these equations assume negligible A and B term[8] MCD intensity)

$$\frac{k_BT}{\mu_BB}\Delta A_{\text{MCD}} = \sum_{n=1}^{N} \underbrace{\gamma c l C_{0,n}}_{=C'_n} \nu \exp\left[-\frac{(\nu-\mu_n)^2}{2\sigma_n^2}\right] = \sum_{n=1}^{N} C'_n \nu \exp\left[-\frac{(\nu-\mu_n)^2}{2\sigma_n^2}\right] \tag{S1}$$

$$A_{\text{Abs}} = \sum_{n=1}^{N} \underbrace{\gamma c l D_{0,n}}_{=D'_n} \nu \exp\left[-\frac{(\nu-\mu_n)^2}{2\sigma_n^2}\right] = \sum_{n=1}^{N} D'_n \nu \exp\left[-\frac{(\nu-\mu_n)^2}{2\sigma_n^2}\right] \tag{S2}$$

where γ is a proportionality factor, c is concentration, l is pathlength, ν is wavenumber, N is the total number of peaks to be fit, n indexes these peaks, μ_n is the wavenumber of the maximum of peak n, σ_n controls the linewidth of peak n, $C_{0,n}$ is the C_0 value of peak n, and $D_{0,n}$ is the D_0 value of peak n. In actuality, composite variables $C'_n = \gamma c l C_{0,n}$ and $D'_n = \gamma c l D_{0,n}$ were used in the fitting procedures to avoid explicit inclusion of γ, c, and l, which each have their own associated uncertainties. The C'_n/D'_n ratios equal $C_{0,n}/D_{0,n}$ ratios because the other constants will cancel upon division. Zeroth and first-order baseline corrections were applied where necessary. If the influence of scattering was seen in the baseline, a simple Rayleigh model of scattering[9] was used to subtract the rising baseline

$$A_{\text{scatter}} = \ln\left(\frac{1}{1-s\lambda^{-4}}\right), \tag{S3}$$

where s is a parameter characterizing the scattering and λ is the wavelength of light.

We have approached our experimental measurements by collecting data on at least three separate samples. For the tungsten(V) chalcogenide complexes, each sample was taken from different synthetic batches. All datasets (except those of $Cu(acac)_2$, vide infra) were analyzed by simultaneously fitting Gaussian lineshapes to the data according to Eqs. (S1) and (S2) using least-squares nonlinear regression in Mathematica 14. Reasonable starting values for parameters were provided by estimation by eye, or by independently fitting values to absorption data without simultaneously fitting the MCD data. Standard uncertainties of the fit were calculated by Mathematica and are indicated in each table of fitted parameters at one standard uncertainty in the final digit(s); for example, $4.321(8) = 4.321 \pm 0.008$ and $4.321(12) = 4.321 \pm 0.012$. The final parameters were then averaged to give the values listed in the main manuscript.

S2.2.1 [Na(THF)$_6$][**1**·O]

Three samples of [Na(THF)$_6$][**1**·O] were prepared in THF (Fig. S1); each sample came from a different batch of compound. The concentrations of the samples were ① 1.0, ② 1.6, and ③ 0.70 mM. Two peaks were modeled in the fit, and the C_0/D_0 ratios from the three samples (Table S1) average to −0.466(29) for the lower energy transition ($E_{1/2}$) and +0.32(4) for the higher energy transition ($E_{3/2}$). For a discussion of the $\Delta\epsilon/2\epsilon$ plots in Figure 2(b) were calculated, see Section S2.4.3.

Table S1: [Na(THF)$_6$][**1**·O] Lineshape Fitting

Sample ①		Sample ②		Sample ③	
Parameter	Fit	Parameter	Fit	Parameter	Fit
D_1'	$1.073(19) \times 10^{-5}$ cm	D_1'	$1.764(16) \times 10^{-5}$ cm	D_1'	$1.025(15) \times 10^{-5}$ cm
C_1'	$-5.28(8) \times 10^{-6}$ cm	C_1'	$-8.42(7) \times 10^{-6}$ cm	C_1'	$-4.39(6) \times 10^{-6}$ cm
μ_1	14883(15) cm^{-1}	μ_1	14814(8) cm^{-1}	μ_1	14916(13) cm^{-1}
σ_1	1186(18) cm^{-1}	σ_1	1207(9) cm^{-1}	σ_1	1210(16) cm^{-1}
D_2'	$1.276(11) \times 10^{-5}$ cm	D_2'	$2.113(9) \times 10^{-5}$ cm	D_2'	$1.052(9) \times 10^{-5}$ cm
C_2'	$4.10(7) \times 10^{-6}$ cm	C_2'	$5.94(6) \times 10^{-6}$ cm	C_2'	$3.86(6) \times 10^{-6}$ cm
μ_2	17680(16) cm^{-1}	μ_2	17689(8) cm^{-1}	μ_2	17691(14) cm^{-1}
σ_2	1338(15) cm^{-1}	σ_2	1376(8) cm^{-1}	σ_2	1282(12) cm^{-1}
$(C_0/D_0)_1$	−0.492(12)	$(C_0/D_0)_1$	−0.477(6)	$(C_0/D_0)_1$	−0.429(9)
$(C_0/D_0)_2$	+0.321(6)	$(C_0/D_0)_2$	+0.2809(30)	$(C_0/D_0)_2$	+0.367(6)

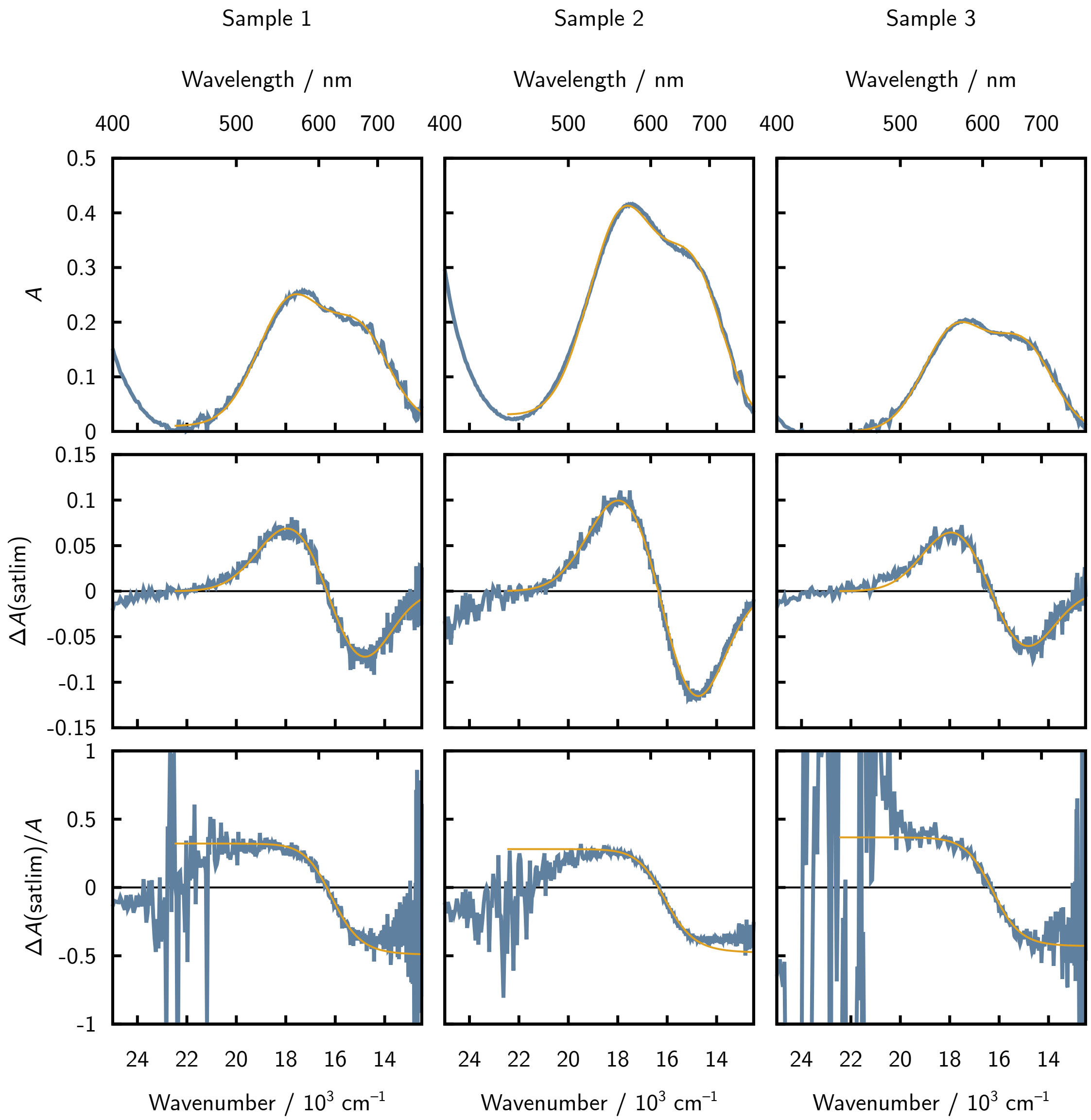

Figure S1: Data for the three $[Na(THF)_6][\mathbf{1}{\cdot}O]$ samples in THF solution. The top row is absorption (A), the middle row is MCD ($\Delta A(\mathrm{satlim}) = (k_B T/\mu_B B)\Delta A$), and the bottom row is the $\Delta A(\mathrm{satlim})/A$ ratio. Blue curves are data and orange curves are fits.

S2.2.2 $[Na(THF)_6][\mathbf{1}{\cdot}S]$

Three samples of $[Na(THF)_6][\mathbf{1}{\cdot}S]$ were prepared in THF (Fig. S1); each sample came from a different batch of compound. The concentrations of the samples were ① 1.3, ② 3.1, and ③ 5.0 mM. Three peaks were modeled in the fit, and the C_0/D_0 ratios from the three samples (Table S2) average to −0.392(32) for the lower energy transition ($E_{1/2}$), +0.443(9) for the middle transition ($E_{3/2}$), and −0.21(4) for the higher energy transition ($E_{1/2}$).

Table S2: $[Na(THF)_6][\mathbf{1}{\cdot}S]$ Lineshape Fitting

Sample ①		Sample ②		Sample ③	
Parameter	Fit	Parameter	Fit	Parameter	Fit
D'_1	$2.264(6) \times 10^{-5}$ cm	D'_1	$5.455(12) \times 10^{-5}$ cm	D'_1	$9.058(21) \times 10^{-5}$ cm
C'_1	$-9.90(5) \times 10^{-6}$ cm	C'_1	$-2.017(10) \times 10^{-5}$ cm	C'_1	$-3.344(19) \times 10^{-5}$ cm
μ_1	7574.2(3.2) cm^{-1}	μ_1	7601.3(2.8) cm^{-1}	μ_1	7619.1(3.1) cm^{-1}
σ_1	934(4) cm^{-1}	σ_1	973.9(3.4) cm^{-1}	σ_1	993(4) cm^{-1}
D'_2	$2.155(6) \times 10^{-5}$ cm	D'_2	$5.237(11) \times 10^{-5}$ cm	D'_2	$8.689(20) \times 10^{-5}$ cm
C'_2	$9.80(5) \times 10^{-6}$ cm	C'_2	$2.268(10) \times 10^{-5}$ cm	C'_2	$3.839(18) \times 10^{-5}$ cm
μ_2	10333.2(3.4) cm^{-1}	μ_2	10387.6(2.9) cm^{-1}	μ_2	10421.1(3.2) cm^{-1}
σ_2	1010.8(3.5) cm^{-1}	σ_2	1018.4(3.0) cm^{-1}	σ_2	1015.3(3.2) cm^{-1}
D'_3	$3.19(4) \times 10^{-6}$ cm	D'_3	$7.71(5) \times 10^{-6}$ cm	D'_3	$1.453(10) \times 10^{-5}$ cm
C'_3	$-8.2(4) \times 10^{-7}$ cm	C'_3	$-1.66(4) \times 10^{-6}$ cm	C'_3	$-2.30(11) \times 10^{-6}$ cm
μ_3	16662(12) cm^{-1}	μ_3	16700(7) cm^{-1}	μ_3	16756(6) cm^{-1}
σ_3	875(17) cm^{-1}	σ_3	888(9) cm^{-1}	σ_3	956(9) cm^{-1}
$(C_0/D_0)_1$	−0.4371(26)	$(C_0/D_0)_1$	−0.3698(21)	$(C_0/D_0)_1$	−0.3692(22)
$(C_0/D_0)_2$	+0.4549(26)	$(C_0/D_0)_2$	+0.4331(21)	$(C_0/D_0)_2$	+0.4418(23)
$(C_0/D_0)_3$	−0.257(12)	$(C_0/D_0)_3$	−0.215(6)	$(C_0/D_0)_3$	−0.159(7)

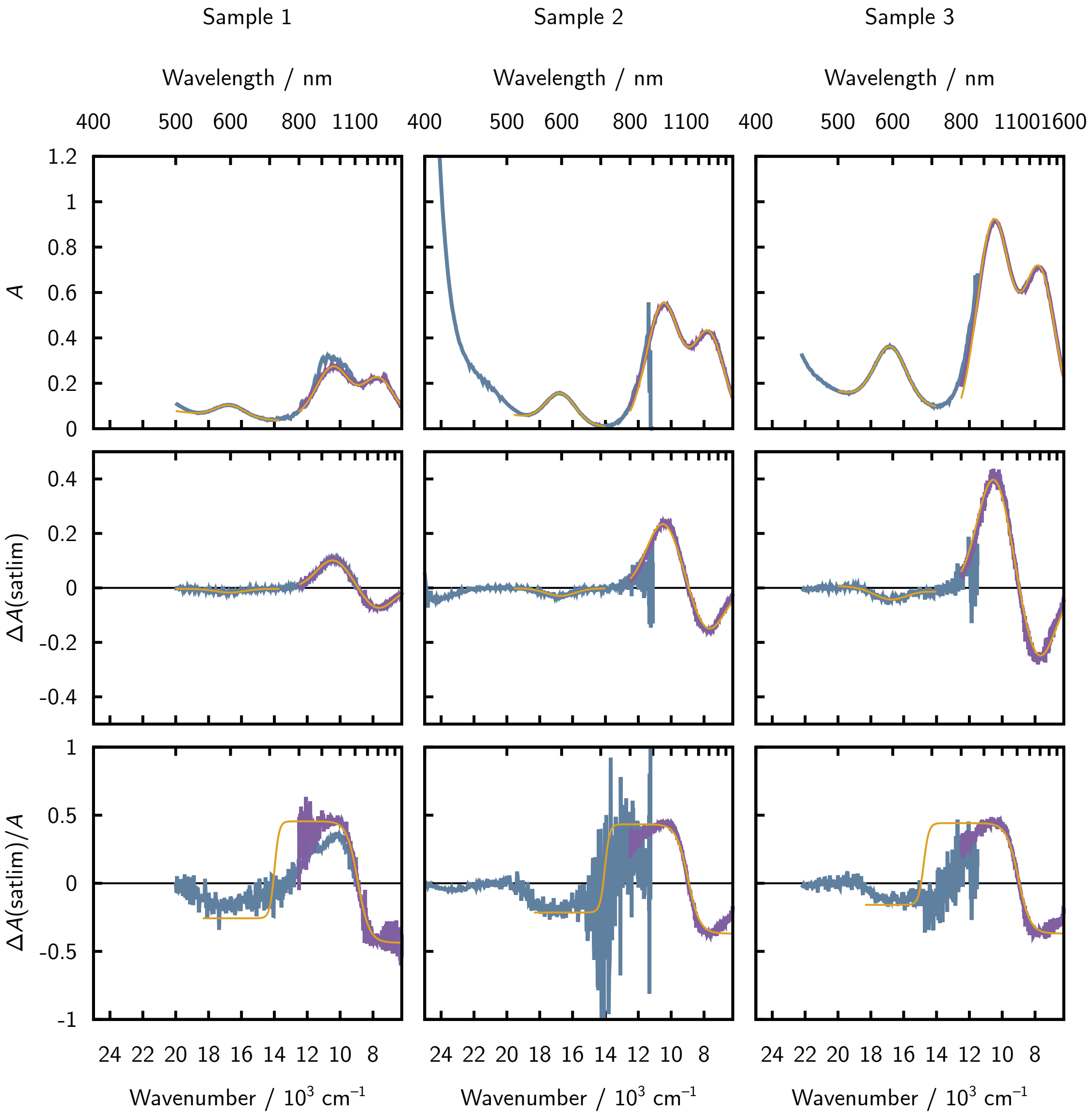

Figure S2: Data for the three $[Na(THF)_6][\mathbf{1}{\cdot}S]$ samples in THF solution. The top row is absorption (A), the middle row is MCD ($\Delta A(\mathrm{satlim}) = (k_B T/\mu_B B)\Delta A$), and the bottom row is the $\Delta A(\mathrm{satlim})/A$ ratio. Blue curves are data acquired using the PMT detector, purple curves are data acquired using the InGaAs detector, and orange curves are fits.

S2.2.3 $[Na(THF)_x(Et_2O)_y][\mathbf{1}\cdot Se]$

Three samples of $[Na(THF)_x(Et_2O)_y][\mathbf{1}\cdot Se]$ were prepared in THF; each sample came from a different batch of compound. The concentrations of the samples were ① 4.3, ② 5.0, and ③ 2.0 mM. Three peaks were modeled in the fit, and the C_0/D_0 ratios from the three samples (Table S3) average to −0.407(15) for the lower energy transition ($E_{1/2}$), +0.429(5) for the middle transition ($E_{3/2}$), and −0.259(8) for the higher energy transition ($E_{1/2}$).

Table S3: $[Na(THF)_x(Et_2O)_y][\mathbf{1}\cdot Se]$ Lineshape Fitting

Sample ①		Sample ②		Sample ③	
Parameter	Fit	Parameter	Fit	Parameter	Fit
D'_1	$5.823(15) \times 10^{-5}$ cm	D'_1	$6.825(17) \times 10^{-5}$ cm	D'_1	$2.510(7) \times 10^{-5}$ cm
C'_1	$-2.417(13) \times 10^{-5}$ cm	C'_1	$-2.855(16) \times 10^{-5}$ cm	C'_1	$-9.69(6) \times 10^{-6}$ cm
μ_1	6525(8) cm^{-1}	μ_1	6549(8) cm^{-1}	μ_1	6526(9) cm^{-1}
σ_1	952(7) cm^{-1}	σ_1	943(7) cm^{-1}	σ_1	970(8) cm^{-1}
D'_2	$5.865(11) \times 10^{-5}$ cm	D'_2	$6.921(13) \times 10^{-5}$ cm	D'_2	$2.529(5) \times 10^{-5}$ cm
C'_2	$2.547(10) \times 10^{-6}$ cm	C'_2	$2.947(12) \times 10^{-5}$ cm	C'_2	$1.077(5) \times 10^{-5}$ cm
μ_2	9517.8(2.7) cm^{-1}	μ_2	9532.5(2.7) cm^{-1}	μ_2	9542.0(2.8) cm^{-1}
σ_2	1043.5(2.6) cm^{-1}	σ_2	1050.4(2.7) cm^{-1}	σ_2	1039.1(2.7) cm^{-1}
D'_3	$8.81(7) \times 10^{-6}$ cm	D'_3	$1.065(7) \times 10^{-5}$ cm	D'_3	
C'_3	$-2.32(6) \times 10^{-6}$ cm	C'_3	$-2.71(6) \times 10^{-6}$ cm	C'_3	
μ_3	15237(8) cm^{-1}	μ_3	15264(7) cm^{-1}	μ_3	
σ_3	925(11) cm^{-1}	σ_3	938(9) cm^{-1}	σ_3	
$(C_0/D_0)_1$	−0.4152(25)	$(C_0/D_0)_1$	−0.4183(25)	$(C_0/D_0)_1$	−0.3862(26)
$(C_0/D_0)_2$	+0.4342(19)	$(C_0/D_0)_2$	+0.4258(19)	$(C_0/D_0)_2$	+0.4257(20)
$(C_0/D_0)_3$	−0.263(7)	$(C_0/D_0)_3$	−0.254(6)		

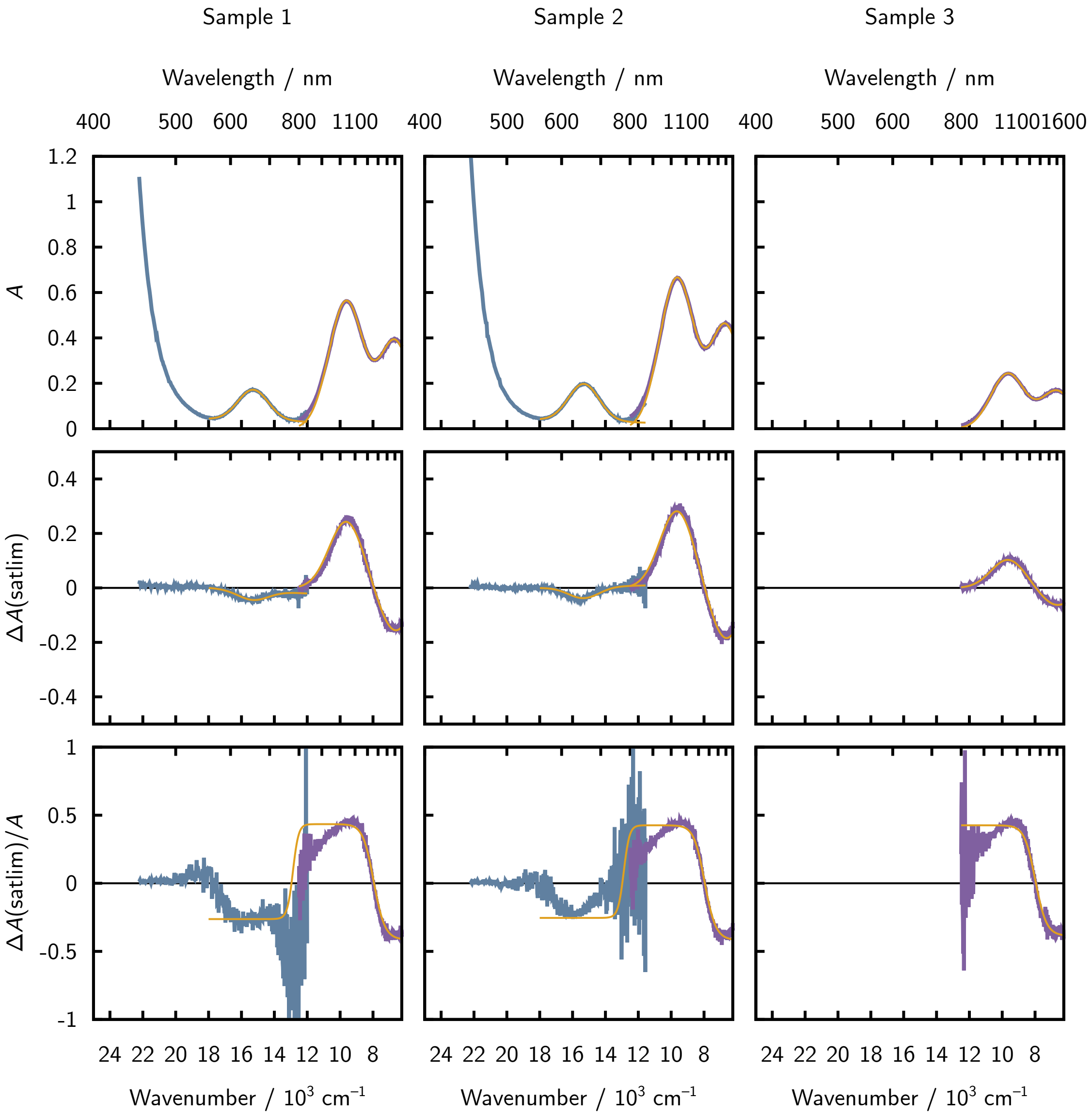

Figure S3: Data for the three $[\mathrm{Na(THF)}_x(\mathrm{Et_2O})_y][\mathbf{1}\cdot\mathrm{Se}]$ samples in THF solution. The top row is absorption (A), the middle row is MCD ($\Delta A(\mathrm{satlim}) = (k_BT/\mu_BB)\Delta A$), and the bottom row is the $\Delta A(\mathrm{satlim})/A$ ratio. Blue curves are data acquired using the PMT detector, purple curves are data acquired using the InGaAs detector, and orange curves are fits.

S2.2.4 TEMPO

Three samples were prepared using commercial (2,2,6,6-tetramethylpiperidin-1-yl)oxyl (TEMPO) dissolved in acetonitrile (Fig. S4). The concentrations of the samples were ① 25, ② 91, and ③ 81 mM. One transition was observed at 456 nm ($\epsilon = 9.9$ M^{-1} cm^{-1}). The C_0/D_0 ratio from the three samples (Table S4) average to 0.040(4).

Table S4: TEMPO Lineshape Fitting

Sample ①		Sample ②		Sample ③	
Parameter	Fit	Parameter	Fit	Parameter	Fit
D'_1	$4.693(11) \times 10^{-4}$ cm	D'_1	$3.933(8) \times 10^{-5}$ cm	D'_1	$3.505(7) \times 10^{-5}$ cm
C'_1	$1.65(8) \times 10^{-5}$ cm	C'_1	$1.68(5) \times 10^{-6}$ cm	C'_1	$1.45(5) \times 10^{-6}$ cm
μ_1	21717(6) cm^{-1}	μ_1	21649(5) cm^{-1}	μ_1	21637(5) cm^{-1}
σ_1	2273(7) cm^{-1}	σ_1	2188(6) cm^{-1}	σ_1	2207(6) cm^{-1}
$(C_0/D_0)_1$	0.0351(18)	$(C_0/D_0)_1$	0.0427(14)	$(C_0/D_0)_1$	0.0414(14)

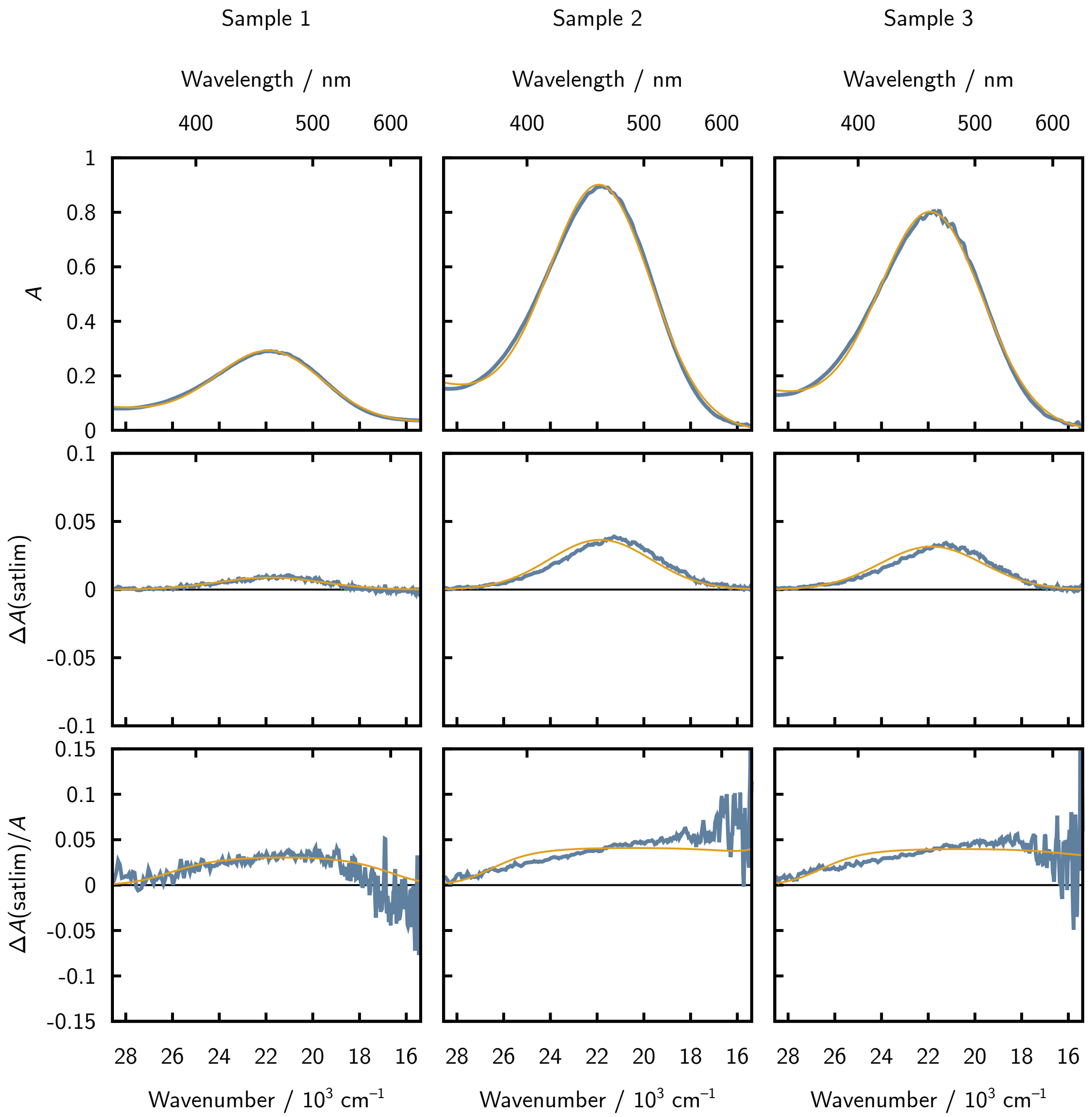

Figure S4: Data for the three TEMPO samples in acetonitrile solution. The top row is absorption (A), the middle row is MCD ($\Delta A(\text{satlim}) = (k_B T/\mu_B B)\Delta A$), and the bottom row is the $\Delta A(\text{satlim})/A$ ratio. Blue curves are data and orange curves are fits.

S2.2.5 Potassium Hexachloroiridate(IV)

The MCD spectrum of the hexachloroiridate(IV) anion has been investigated several times, and a thorough discussion can be found in the excellent treatise on group theory and MCD spectroscopy by Piepho and Schatz (Sec. 23.2 "A Favorite Example: $IrCl_6^{2-}$").[10,11] As explained in the paper by Sutcliffe et al., the MCD spectrum in the 350–650 nm region has three clear bands, each of which is expected to exhibit fine structure from SOC splitting. The classic treatments of $[IrCl_6]^{2-}$ in solution by Piepho and Schatz[10] and by Henning et al.[11] do not treat the shoulders that can be seen on bands II and III because the SOC was expected to be smaller than the linewidth of the transitions. Despite the visible splittings in our spectra, we have followed the previous analyses of these authors and have fit a single Gaussian lineshape to each band; in this way, our fit provides an 'average' C_0/D_0 ratio across each band. Data were collected on three samples (Fig. S5). The concentrations of the three samples were ① 2.0 mM (0.1 cm path length cuvette), ② 0.51, and ③ 0.36 mM. The results are given in Table S5, and give average C_0/D_0 ratios of 0.26(6), 0.649(17), and −0.678(12) for bands I (578 nm), II (490 nm), and III (430 nm). Our values in aqueous solution are slightly smaller than those of Henning et al. in dichloroethane solution: 0.90 for Band II and −0.82 for Band III (see Henning et al. for the original data, and see Piepho & Schatz for a conversion into the modern conventional definitions of C_0 and D_0).[10,11]

Table S5: Aqueous K_2IrCl_6 Lineshape Fitting

Sample ①		Sample ②		Sample ③	
Parameter	Fit	Parameter	Fit	Parameter	Fit
D'_1	$3.60(19) \times 10^{-6}$ cm	D'_1	$1.05(17) \times 10^{-5}$ cm	D'_1	$7.7(1.3) \times 10^{-6}$ cm
C'_1	$1.05(6) \times 10^{-6}$ cm	C'_1	$2.6(6) \times 10^{-6}$ cm	C'_1	$-1.8(4) \times 10^{-6}$ cm
μ_1	17265(28) cm^{-1}	μ_1	17306(90) cm^{-1}	μ_1	17300(91) cm^{-1}
σ_1	971(51) cm^{-1}	σ_1	946(167) cm^{-1}	σ_1	940(169) cm^{-1}
D'_2	$2.510(13) \times 10^{-5}$ cm	D'_2	$8.00(12) \times 10^{-5}$ cm	D'_2	$5.84(9) \times 10^{-5}$ cm
C'_2	$1.675(8) \times 10^{-5}$ cm	C'_2	$5.10(7) \times 10^{-5}$ cm	C'_2	$3.76(5) \times 10^{-5}$ cm
μ_2	20386.4(2.4) cm^{-1}	μ_2	20407(7) cm^{-1}	μ_2	20400(7) cm^{-1}
σ_2	713.3(2.6) cm^{-1}	σ_2	699(8) cm^{-1}	σ_2	700(8) cm^{-1}
D'_3	$1.837(9) \times 10^{-5}$ cm	D'_3	$5.77(9) \times 10^{-5}$ cm	D'_3	$4.21(6) \times 10^{-5}$ cm
C'_3	$-1.247(6) \times 10^{-5}$ cm	C'_3	$-3.89(5) \times 10^{-5}$ cm	C'_3	$-2.87(4) \times 10^{-5}$ cm
μ_3	23402(4) cm^{-1}	μ_3	23422(13) cm^{-1}	μ_3	23417(13) cm^{-1}
σ_3	1230(5) cm^{-1}	σ_3	1220(16) cm^{-1}	σ_3	1220(16) cm^{-1}
$(C_0/D_0)_1$	+0.291(23)	$(C_0/D_0)_1$	+0.24(7)	$(C_0/D_0)_1$	−0.23(7)
$(C_0/D_0)_2$	+0.668(5)	$(C_0/D_0)_2$	+0.637(3)	$(C_0/D_0)_2$	+0.643(14)
$(C_0/D_0)_3$	−0.679(5)	$(C_0/D_0)_3$	−0.674(14)	$(C_0/D_0)_3$	−0.681(14)

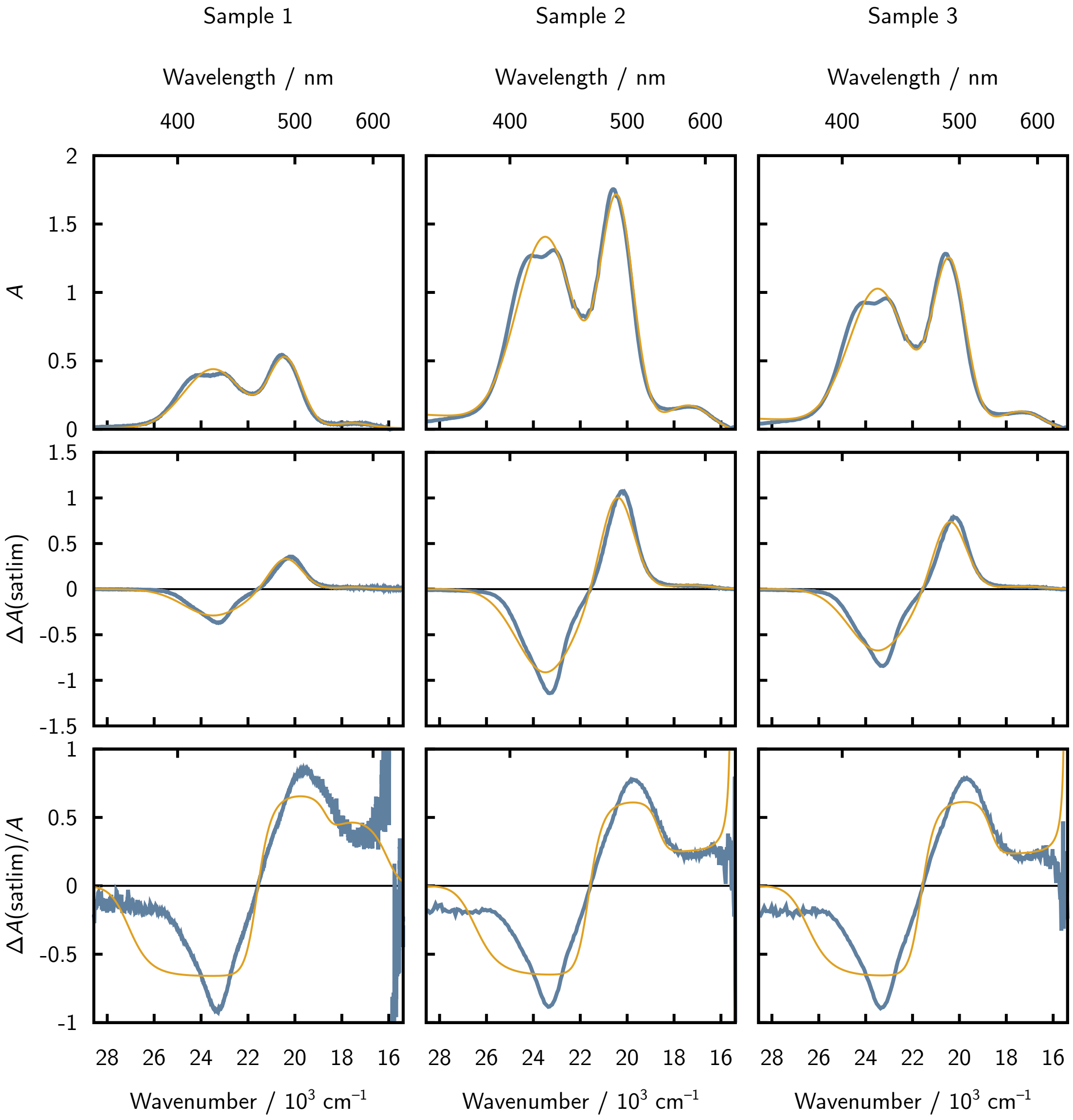

Figure S5: Data for the three aqueous K_2IrCl_6 samples. The top row is absorption (A), the middle row is MCD ($\Delta A(\text{satlim}) = (k_B T/\mu_B B)\Delta A$), and the bottom row is the $\Delta A(\text{satlim})/A$ ratio. Blue curves are data and orange curves are fits. There is clear splitting in the 420 nm feature that this model does not capture, but we have chosen to use this model because it matches the one used in the thorough treatment of $[IrCl_6]^{2-}$ by Piepho and Schatz.[10]

S2.2.6 Copper(II) Acetylacetonate

Three samples were prepared using commercial copper(II) acetylacetonate $Cu(acac)_2$ dissolved in dichloromethane (Fig. S6). The concentrations of the samples were ① 12, ② 24, and ③ 18 mM. Fitting of spectra for $Cu(acac)_2$ were made especially complicated due to the strong overlap of four peaks in the 450–800 nm region.[12,13] Fitted parameters showed strong correlation coefficients between many of the parameters such that the standard uncertainties on the resulting C_0/D_0 ratios were unacceptably large. Instead, C_0/D_0 ratios were roughly estimated at a series of wavelengths chosen to lie near the maximum and the minimum of the MCD spectra: 720/730/740 nm were near the maximum and 500/510/520 nm were near the minimum. The C_0/D_0 ratios calculated from the raw data at these wavelengths are summarized in Table S6. The average C_0/D_0 ratios were +0.081(15) around 730 nm and −0.134(14) around 510 nm. Unfortunately, these values are highly sensitive to the particular wavelength, as can be seen by comparing the values at 500, 510, and 520 nm for Sample ②. Such differences can arise from other MCD intensity mechanisms (such as the A term intensity mechanism), from errors in absorption baselines, from the strong overlaps of peaks, from taking ratios of small numbers subject to noise, and more. Nonetheless, we feel the C_0/D_0 ratios from this treatment of the spectra paints a useful picture of the relative C_0/D_0 ratios for comparative purposes.

Table S6: C_0/D_0 Ratios for $Cu(acac)_2$

λ / nm	Sample ①	Sample ②	Sample ③
500	−0.1481	−0.1402	−0.1354
510	−0.1515	−0.1223	−0.1249
520	−0.1420	−0.1076	−0.1297
720	+0.0618	+0.0588	+0.0975
730	+0.0825	+0.0717	+0.0814
740	+0.0811	+0.0918	+0.1013

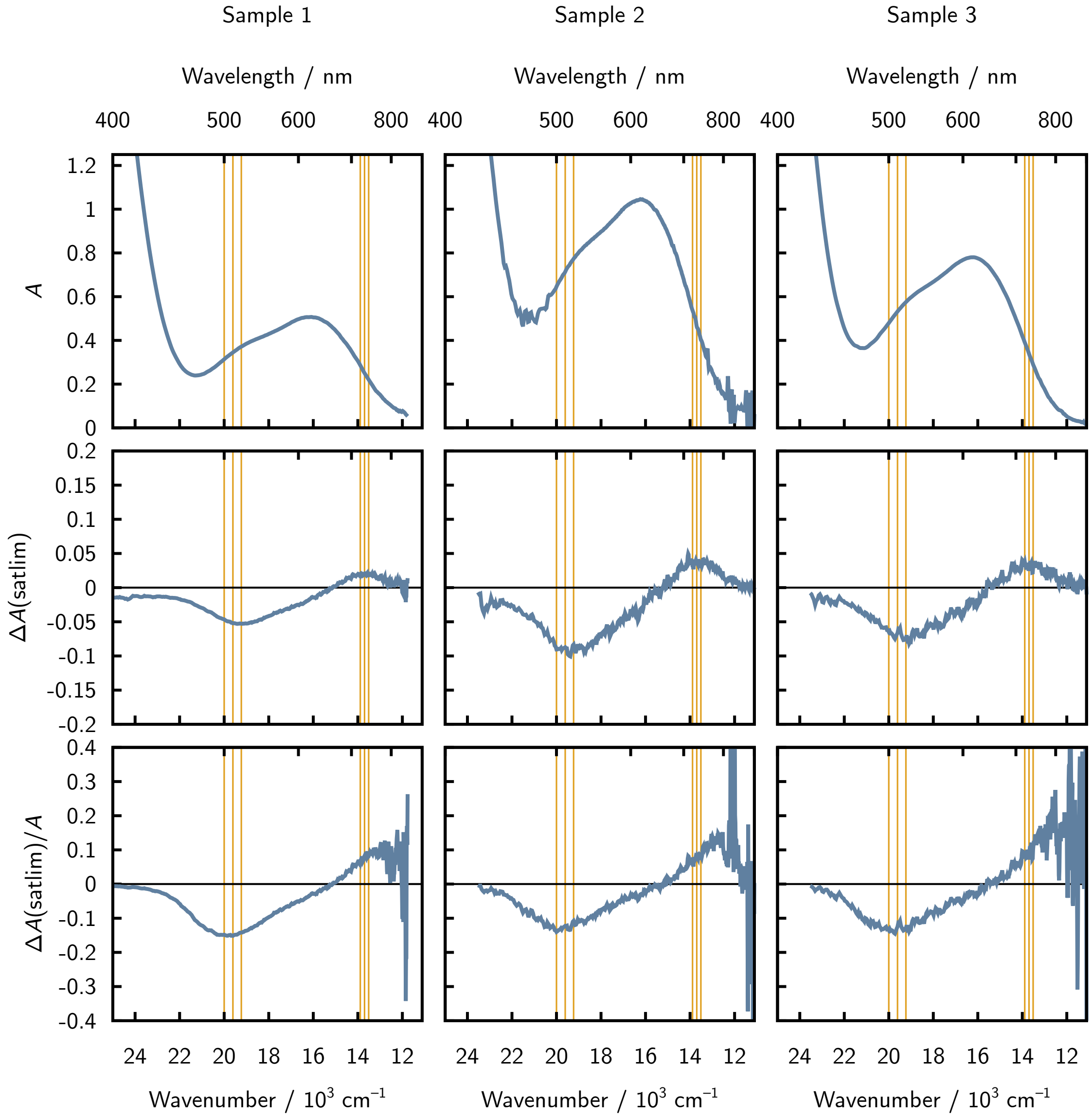

Figure S6: Data for the three $Cu(acac)_2$ samples in dichloromethane solution. The top row is absorption (A), the middle row is MCD ($\Delta A(\text{satlim}) = (k_BT/\mu_BB)\Delta A$), and the bottom row is the $\Delta A(\text{satlim})/A$ ratio. Blue curves are data and orange vertical lines indicate positions where the C_0/D_0 ratios were estimated as described in Sec. S2.2.6.

S2.3 Cryogenic MCD Studies of [Na(THF)$_6$][1·O]

A sample of ∼1 mM concentration in 1:1 THF/toluene was prepared and flash frozen as described in Sec. S2.1. An impurity was seen to leach from the polymer O-ring that caused the growth of a sharp feature at 779 nm, indicated with an asterisk (*) in the plots below. The MCD spectrum projected to the saturation limit (Figure S7) was extracted from the data using a similar pseudoinverse technique as described in Section S2.1:

$$\begin{pmatrix} 1 & \tanh(g\mu_B B_1/2k_B T_1) \\ 1 & \tanh(g\mu_B B_2/2k_B T_2) \\ 1 & \tanh(g\mu_B B_3/2k_B T_3) \\ \vdots & \vdots \end{pmatrix}^{\ominus} \begin{pmatrix} I^{B_1,T_1}_{400\text{ nm}} & I^{B_1,T_1}_{401\text{ nm}} & \cdots & I^{B_1,T_1}_{800\text{ nm}} \\ I^{B_2,T_2}_{400\text{ nm}} & I^{B_2,T_2}_{401\text{ nm}} & \cdots & I^{B_2,T_2}_{800\text{ nm}} \\ I^{B_3,T_3}_{400\text{ nm}} & I^{B_3,T_3}_{401\text{ nm}} & \cdots & I^{B_3,T_3}_{800\text{ nm}} \\ \vdots & \vdots & & \vdots \end{pmatrix} = \begin{pmatrix} I^{\text{baseline}}_{400\text{ nm}} & \cdots & I^{\text{baseline}}_{800\text{ nm}} \\ I^{\text{satlim}}_{400\text{ nm}} & \cdots & I^{\text{satlim}}_{800\text{ nm}} \end{pmatrix},$$

where the leftmost matrix now contains a column indicating constant baseline between spectra and a second column indicating the fractional magnetization of the sample. Due to the single-beam nature of the JASCO J-1700 spectrometer, the absorption data are far noisier than the MCD data, and the absorption spectrum shows some artifacts from the Xe arc lamp that arise from imperfect subtraction of the background. MCD spectra were acquired every 1 T up to a field strength 7 T, all at a temperature of 5 K. For a discussion of how the $\Delta\epsilon/2\epsilon$ plots in Figure 2(b) were calculated, see Section S2.4.3.

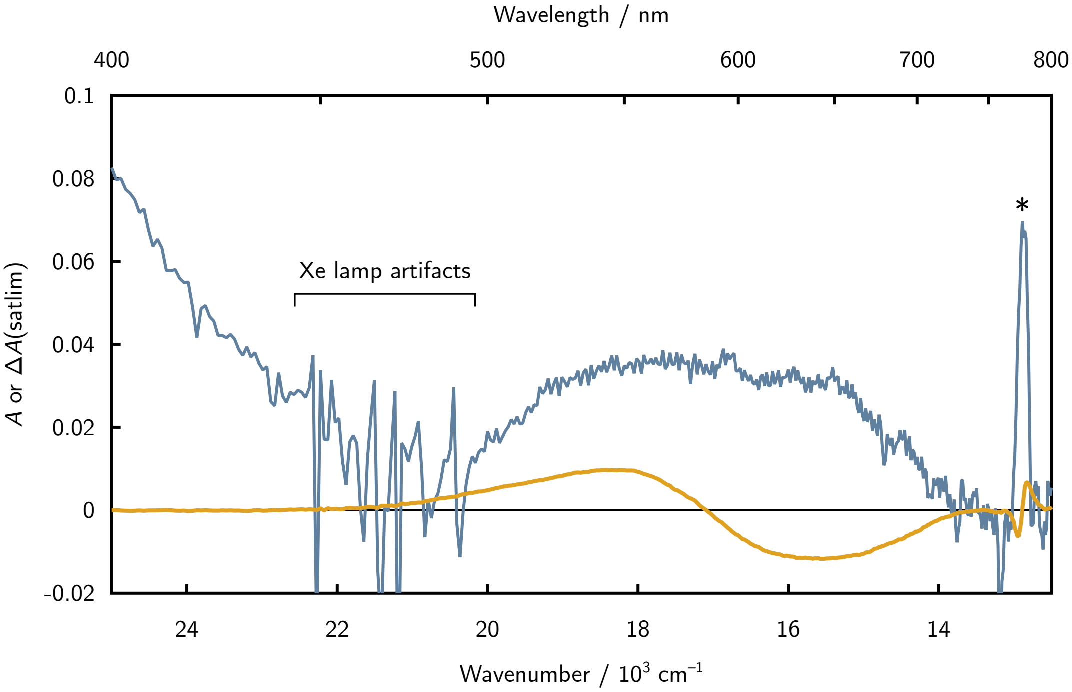

Figure S7: Absorption spectrum and MCD spectrum (projected to the saturation limit) of [Na(THF)$_6$][**1**·O] in a frozen 1:1 THF/toluene glass at 5 K.

S2.4 Theoretical Considerations

MCD spectroscopy measures the differential absorptivity ($\Delta\epsilon$) of LCP (left circularly polarized) and RCP (right circularly polarized) light of a sample in a magnetic field,

$$\Delta\epsilon = \epsilon_{\mathrm{LCP}} - \epsilon_{\mathrm{RCP}}. \tag{S4}$$

In the high temperature and/or low field limit, the MCD intensity is usually described in a linearized form using three "Faraday terms" A_1, B_0, and C_0,[8,10]

$$\frac{\Delta\epsilon}{E} = \gamma\mu_B B\left[A_1\left(-\frac{\partial f(E)}{\partial E}\right) + \left(B_0 + \frac{C_0}{k_B T}\right) f(E)\right], \tag{S5}$$

where each Faraday term parametrizes a particular intensity mechanism. Here, E is the energy of measurement, γ a proportionality constant,* μ_B the Bohr magneton, B the applied magnetic field, k_B the Boltzmann constant, T the temperature, and $f(E)$ a lineshape function. The dichroic response induced by the applied field is related to the unpolarized absorptivity through

$$\epsilon = \frac{\epsilon_{\mathrm{LCP}} + \epsilon_{\mathrm{RCP}}}{2}, \tag{S6}$$

where the factor of two in the denominator arises because unpolarized absorption will appear as the average of absorption using polarized light. A fourth parameter D_0 is usually introduced to describe the absorption intensity,

$$\frac{\epsilon}{E} = \frac{\gamma}{2} D_0 f(E) \tag{S7}$$

and this definition allows facile comparison of MCD and absorptivity through C_0/D_0 ratios.

S2.4.1 Predicting Signs of C Term Intensity Using the Wigner–Eckart Theorem

We use the Altmann point groups tables for all double group treatments because they provide clear matrix representatives and Clebsch–Gordan coefficients.[14]

The general MCD intensity equation Eq. (S8) shows that MCD is sensitive to the populations of the ground and excited states, the transition dipole matrix elements connecting these states, and the lineshape associated with the transition:

$$\frac{\Delta\epsilon}{E} = \gamma\sum_{\substack{a\in A\\ j\in J}}(N_a - N_j)\Big(\underbrace{|\langle Aa|m_-|Jj\rangle|^2}_{\text{LCP}} - \underbrace{|\langle Aa|m_+|Jj\rangle|^2}_{\text{RCP}}\Big) f_{a\to j}(E), \tag{S8}$$

where a indexes levels within the ground state, j indexes levels within the excited state, N_p is the fractional population of level p, $m_\pm = (m_x \pm i m_y)/\sqrt{2}$ the transition dipole operators, and $f_{p\to q}(E)$ a lineshape function between levels p and q. Assuming negligible excited state population ($N_j \approx 0$) and using the rigid-shift approximation,[10] we obtain an expression for C term intensity,

$$\frac{\Delta\epsilon}{E} \approx \gamma\sum_{\substack{a\in A\\ j\in J}} N_a\left(|\langle Aa|m_-|Jj\rangle|^2 - |\langle Aa|m_+|Jj\rangle|^2\right) f(E) \tag{S9}$$

The γ, N_a, and $f(E)$ values must always be positive, so the sign of C term intensity results exclusively from the difference in LCP/RCP dipole operator matrix elements.

The Wigner–Eckart theorem (WET) helps to evaluate matrix elements in the presence of symmetry. If irreducible representations (irreps) of the point/double group can be associated with the bra, operator, and ket of a matrix element, $\langle\Phi|O|\Psi\rangle \to \langle\Phi^a_\alpha|O^f_\phi|\Psi^b_\beta\rangle$, then the WET can be used. Here, $a/f/b$ indicate irreps of

*The constant γ has been defined in a couple of ways. Piepho & Schatz[10] define γ as the proportionality constant for $\Delta A/E$ such that $\gamma_{\mathrm{P\&S}} = \gamma_{\mathrm{Here}} cl$, where c is concentration and l is path length.

the group, and $\alpha/\phi/\beta$ are the respective components within irreps $a/f/b$. In non-isometric point groups,[†] this theorem takes the form[10]

$$\langle \Phi^a_\alpha | O^f_\phi | \Psi^b_\beta \rangle = \langle \Phi^a \| O^f \| \Psi^b \rangle (f\phi, b\beta | a\alpha)^*, \tag{S10}$$

where $\langle \Phi^a \| O^f \| \Psi^b \rangle$ is called a 'reduced matrix element' that does not depend on the components of the irreps, and $(f\phi, b\beta|a\alpha)$ is a Clebsch–Gordan coefficient (CGC). There are unfortunately a wide number of notations used to express CGCs, and our $(f\phi, b\beta|a\alpha)$ notation above is equivalent to $\left(\begin{smallmatrix} f & b \\ \phi & \beta \end{smallmatrix}\middle|\begin{smallmatrix} a \\ \alpha \end{smallmatrix}\right)$, $\langle f\phi, b\beta|(fb)a\alpha\rangle$, ${}^{fb}\langle \phi\beta|a\alpha\rangle$, $C^{a\alpha}_{f\phi b\beta}$, etc.

We can use this theorem to predict the signs of MCD C term intensity for the ${}^2B_2 \to {}^2E$ transitions of pseudo-C_{4v} $[\mathbf{1}\cdot\mathrm{E}]^-$ complexes, but to do so we first need to find the symmetries of the bras, operators, and kets. Because MCD depends on the spin M_S levels of the ground state, we must use C^*_{4v} double group irreps. We use the Altmann tables,[14] which indicate that a $S = 1/2$ spin transforms as $E_{1/2}$, and within this irrep that $|M_S = +1/2\rangle$ transforms as component 1 and $|M_S = -1/2\rangle$ as component 2. The ground state ${}^2B_2$ thus transforms as $E_{1/2} \otimes B_2 = E_{3/2}$ and we obtain symmetry-adapted wavefunctions:

$$\begin{aligned} |E_{3/2}1({}^2B_2)\rangle &= \sum_\alpha (E_{1/2}\alpha, B_2|E_{3/2}1)\, |E_{1/2}\alpha\rangle|B_2\rangle = |E_{1/2}1\rangle|B_2\rangle = |{}^2B_2, M_S = +1/2\rangle \\ |E_{3/2}2({}^2B_2)\rangle &= \sum_\alpha (E_{1/2}\alpha, B_2|E_{3/2}2)\, |E_{1/2}\alpha\rangle|B_2\rangle = -|E_{1/2}2\rangle|B_2\rangle = -|{}^2B_2, M_S = -1/2\rangle \end{aligned} \tag{S11}$$

Next, we find symmetry-adapted operators. The ${}^2B_2 \to {}^2E$ transitions are x, y polarized so we can assume the magnetic field is directed along the molecular z axis. The symmetrized bases listed by Altmann show

$$\begin{aligned} m^E_1 &= m_+ \\ m^E_2 &= -m_- \end{aligned} \tag{S12}$$

Lastly, we need symmetry-adapted wavefunctions for the excited state, which splits as $E_{1/2} \otimes E = E_{1/2} + E_{3/2}$. The principle of spectroscopic stability[10] allows us to work directly in the excited state symmetrized basis without solving for correlations to the unsymmetrized wavefunctions.

Application of a positive magnetic field along z will cause the $M_S = -1/2$ level to drop in energy, leading to excess population. Thus, this is the level that will determine the C term MCD sign. Eq. (S9) will be positive if

$$\sum_{j\in J} |\langle Aa|m_-|Jj\rangle|^2 > \sum_{j\in J} |\langle Aa|m_+|Jj\rangle|^2 \tag{S13}$$

and negative otherwise. Let's treat the $E_{1/2}$ portion of the ${}^2E$ excited state first. We can adapt this inequality to work directly with symmetry-adapted wavefunctions and operators:

$$\sum_{j=1}^{2} \left|-\langle E_{3/2}2({}^2B_2)|(-m^E_2)|E_{1/2}j({}^2E)\rangle\right|^2 \overset{?}{>} \sum_{j=1}^{2} \left|-\langle E_{3/2}2({}^2B_2)|(m^E_1)|E_{1/2}j({}^2E)\rangle\right|^2 \tag{S14}$$

Using the WET, this becomes

$$\begin{aligned} &\left|\langle E_{3/2}({}^2B_2)\|m^E\|E_{1/2}({}^2E)\rangle\right|^2 \sum_{j=1}^{2} \left|(E2, E_{1/2}j|E_{3/2}2)^*\right|^2 \\ &\overset{?}{>} \left|\langle E_{3/2}({}^2B_2)\|m^E\|E_{1/2}({}^2E)\rangle\right|^2 \sum_{j=1}^{2} \left|(E1, E_{1/2}j|E_{3/2}2)^*\right|^2 \end{aligned} \tag{S15}$$

[†] Isometric (tetrahedral, octahedral, and icosahedral) groups have additional complications whenever $f \otimes b$ has a repeated irrep in the product. For example, in the I_h point group, $G_g \otimes H_u = T_{1u} \oplus T_{2u} \oplus G_u \oplus 2H_u$ and the presence of two H_u irreps in the direct product requires that each H_u product irrep be treated separately and carefully.

and division through by the (presumably nonzero) reduced matrix element, we see that the sign of the MCD C term depends exclusively on symmetry (i.e. on the CGCs):

$$\sum_{j=1}^{2} \left|(E2, E_{1/2}j|E_{3/2}2)^*\right|^2 \overset{?}{>} \sum_{j=1}^{2} \left|(E1, E_{1/2}j|E_{3/2}2)^*\right|^2 \tag{S16}$$

When we evaluate these CGCs, we get

$$(0)^2 + (0)^2 \not> (-1)^2 + (0)^2 \tag{S17}$$

thus the $E_{3/2}(^2B_2) \to E_{1/2}(^2E)$ transition has a *negative* MCD feature. Repetition for the $E_{3/2}$ portion of the ^2E excited state shows it must give a *positive* MCD feature if purely x, y polarized.

S2.4.2 MCD Saturation Behavior and Maximal $|C_0/D_0|$ Ratios

The C term MCD intensity expression Eq. (S9) generally predicts a nonlinear response with field because the fractional population N_a is nonlinear. This nonlinear behavior is apparent when the sample experiences some degree of magnetic saturation, leading to complicated behavior of MCD intensity that can be used to gain insight into the splitting within the ground state.[15–18] However, simple $S = 1/2$ systems that are well described by a spin Hamiltonian have a predictable behavior when experiencing saturation, which has been expressed as[15,19]

$$\frac{\Delta\epsilon}{E} = A_{\text{satlim}} \tanh\left(\frac{g\mu_B B}{2k_B T}\right) f(E). \tag{S18}$$

At high temperature and/or low fields ($\mu_B B/k_B T \ll 1$), the hyperbolic tangent function can be approximated at first-order as $\tanh x \approx x + O(x^2)$, recovering a linearized form

$$\frac{\Delta\epsilon}{E} = A_{\text{satlim}} \frac{g\mu_B B}{2k_B T} f(E). \tag{S19}$$

Equating this expression with the C term portion of Eq. (S5), $\Delta\epsilon/E = \gamma\mu_B B(C_0/k_B T)f(E)$, allows us to see $A_{\text{satlim}} = 2\gamma C_0/g$. This gives the expression from the main manuscript,

$$\frac{\Delta\epsilon}{E} = \gamma\frac{2C_0}{g} \tanh\left(\frac{g\mu_B B}{2k_B T}\right) f(E). \tag{1}$$

In the case of $[\mathbf{1}{\cdot}\mathrm{E}]^-$ (E = O, S, Se), the $^2B_2 \to {}^2E$ transitions are x, y-polarized, so it is appropriate to use the $g_{\|}$ (g_{zz}) value in this equation. More complicated expressions can be derived that account more accurately for g anisotropy, but use of g_{iso} should serve as a good approximation for simple $S = 1/2$ systems without large g anisotropies if the appropriate g value to use is unclear.

Eqs. (S4) and (S6) reveal that the maximum magnitude of $\Delta\epsilon/\epsilon$ is ± 2, and this can be used to establish a maximum value for C_0 for systems well described by the treatment above. In the limit of complete saturation, Eq. (1) indicates the maximum MCD C term intensity,

$$\lim_{\frac{\mu_B B}{k_B T}\to\infty} \left(\frac{\Delta\epsilon}{E}\right) = \gamma\frac{2C_0}{g} f(E), \tag{S20}$$

and the ratio of this and Eq. (S7) provides an expression relating $\Delta\epsilon/\epsilon$ and C_0/D_0 in the saturation limit,

$$\lim_{\frac{\mu_B B}{k_B T}\to\infty} \left(\frac{\Delta\epsilon/E}{\epsilon/E}\right) = \frac{\gamma(2C_0/g)f(E)}{\gamma D_0 f(E)/2} = \frac{4C_0}{gD_0}. \tag{S21}$$

Using the maximum value of ± 2 for $\Delta\epsilon/\epsilon$, we get bounds on the value of the C_0/D_0 ratio,

$$\pm 2 = \frac{4C_0}{gD_0} \quad \Rightarrow \quad \frac{C_0}{D_0} = \pm\frac{g}{2}. \tag{S22}$$

S2.4.3 Maximum Spin Polarization through OIM and Figure 2(b)

Assuming the degree of spin polarization is entirely controlled by the efficiency of OIM, the theoretical limit for OIM-driven spin polarization occurs when $\epsilon_{\mathrm{LCP}} > 0$ and $\epsilon_{\mathrm{RCP}} = 0$ for one M_S level, and $\epsilon_{\mathrm{LCP}} = 0$ and $\epsilon_{\mathrm{RCP}} > 0$ for the other M_S level. For a hypothetical molecule exhibiting these properties, Eqs. (S4) and (S6) show $\Delta\epsilon/2\epsilon = \pm 1$ in the saturation limit. Sustained irradiation with LCP (or RCP) light should bleach just one M_S level (assuming electronic relaxation does not also perfectly preserve M_S), allowing asymptotic approach towards 100% spin polarization. In a more realistic system, $|\Delta\epsilon/2\epsilon| < 1$ and the equilibrium spin polarization upon sustained LCP/RCP irradiation will additionally be sensitive to the lifetime of the electronic excited state and the spin–lattice T_1 time. The theoretical OIM limit of $|\Delta\epsilon/2\epsilon|$ would then decrease from unity.

Practically, experimental achievement of the maximum spin polarization of a system through OIM is limited by the same considerations as other optical spin polarization methods, particularly photodamage and photochemistry. An additional consideration is the lifetime of the electronic/optical excited state T_{opt}, which is important when choosing whether to pursue spin polarization within the ground state or the excited state. There are two limiting cases. Case (1): If the electronic lifetime is far shorter than the spin lifetime ($T_{\mathrm{opt}} \ll T_1$) then spin polarization can be expected to accumulate in the ground state of the system. Systems like tetraarylchromium(IV) qubits[20] and $[\mathrm{Cu(H_2O)_6}]\mathrm{SO_4/K_2IrCl_6}$[21,22] generally fall into Case (1). Case (2): If the electronic lifetime is far longer than the spin lifetime ($T_{\mathrm{opt}} \gg T_1$) then spin polarization can be pursued within the excited state, and the T_{opt} lifetime will limit how rapidly OIM can be repeated for a system. Spin-correlated radical pair (SCRP) qubits[23,24] generally fall into Case (2), as it is the dynamics between singlet and triplet excited states that cause spin polarization. For systems in Case (2), it is also important that T_{opt} exceed the standard microwave manipulation time in order to enable practical implementations of delay-after-flash EPR experiments using OIM.

Figure 2(b) of the main manuscript estimates the theoretical OIM limit $\Delta\epsilon_{\mathrm{satlim}}/2\epsilon$ in two ways, depicted through a red and purple curve.

The red data were obtained using the experimental MCD spectrum acquired at room temperature (294 K, 1.5 T) and the absorption spectrum from the same sample. The MCD data were scaled to the saturation limit using a factor of k_BT/μ_BB to estimate $\Delta\epsilon_{\mathrm{satlim}}$. The ratio $\Delta\epsilon_{\mathrm{satlim}}/2\epsilon$ acquired in this way is plotted as a function of wavelength, showing that the theoretical maximum for OIM-driven spin polarization will vary by wavelength of excitation. The dotted red line comes from the Gaussian lineshape fitting that was performed to acquire C_0/D_0 ratios (see Sec. S2.2) and the percentages listed at the ends of the curves come from these lines of best fit. To be clear, we mean that the dotted line uses Eqs. (S1) and (S2) to plot

$$\frac{\Delta\epsilon_{\mathrm{satlim}}}{2\epsilon} = \frac{\sum_{n=1}^{2} C'_n\, \nu \exp\left[-\frac{(\nu-\mu_n)^2}{2\sigma_n^2}\right]}{2\sum_{n=1}^{2} D'_n\, \nu \exp\left[-\frac{(\nu-\mu_n)^2}{2\sigma_n^2}\right]} \tag{S23}$$

using the definitions of parameters described in Sec. S2.2 and the best fit values tabulated in Table S1. Figure 2(b) shows the average of the data and the average of the lines of best fit for all three samples of $[\mathrm{Na(THF)_6}][\mathbf{1}\cdot\mathrm{O}]$. The values of +16% and –23% were taken from Eq. (S23) at 500 and 800 nm, respectively.

The purple data were obtained using the experimental MCD spectra acquired under cryogenic conditions (5 K, 7 T). At such a low temperature and such a large magnetic field, the system experiences a large degree of magnetic saturation, which we can estimate to be roughly

$$\tanh\left(\frac{g\mu_BB}{2k_BT}\right) = 66.8\% \tag{S24}$$

using $g = 1.7151$ (see Sec. S3.1 and Table S7), $B = 7$ T, and $T = 5$ K. The MCD spectrum projected to the saturation limit (Section S2.3) was divided by twice the 5 K absorption spectrum to give the purple data. The lineshapes observed in the cryogenic data were not as well approximated by a Gaussian profile (see Sec. S2.3), so the dotted line shown comes from fitting Eq. (S23) directly to the $\Delta A_{\mathrm{2K/7T}}/2A_{\mathrm{2K}}$ data. The values of +14% and –19% are taken from the values of the fit at 500 and 800 nm, respectively.

S3 Electron Paramagnetic Resonance

S3.1 Continuous Wave EPR Experiments

CW-EPR experiments were performed with a Bruker E580 X-Band FT/CW spectrometer equipped with an ER4118X-MD5 resonator. The temperature was kept constant at 80 K using an Oxford ITC503 temperature controller, a CF935 dynamic continuous-flow cryostat, and a LLT 650 low loss transfer tube. We collected 1024 data points over a sweep width of 2000 G. We used a microwave attenuation of 40 dB. We used a modulation frequency of 100 kHz, a modulation amplitude of 5 G, a time constant of 10.24 ms, and a conversion time of 20.48 ms. Each spectrum was collected for 50–200 scans, depending on the sample.

Table S7: EasySpin Fits

Parameter	Oxide	Sulfide	Selenide
`S`	0.5	0.5	0.5
`g`	1.8007 ⊥<br>1.7151 ∥	1.6765 *xx*<br>1.6698 *yy*<br>1.6208 *zz*	1.6400 *xx*<br>1.6289 *yy*<br>1.5952 *zz*
`gStrain`	0.00326 ⊥<br>0[a] ∥	0.0380 *xx*<br>0.00856 *yy*<br>0[a] *zz*	0.0392 *xx*<br>0.0173 *yy*<br>0[a] *zz*
`Nucs`	‘W’	‘W’	‘W’
`A`	229.1 ⊥<br>413.2 ∥	36.7 *xx*<br>215.9 *yy*<br>388.5 *zz*	0[a] *xx*<br>211.0 *yy*<br>412.6 *zz*
`lw`	1.415	2.862	3.507

[a] Constrained to be 0 due to near-unity (>0.99) correlation coefficient with another variable.

Spectra were simulated using the `pepper` function in the EasySpin software.[25] EPR spectrum fitting is a nonlinear regression problem and requires well-informed initial guesses for each variable describing the system; these initial values were obtained manually by inspection. Least-squares fitting was performed using the Nelder–Mead simplex algorithm in three steps: data were first fit using an ‘integral’ target (fitting to the antiderivative), then an ‘as-is’ target, and finally a ‘derivative’ target. Once a fit was obtained, the correlation matrix was inspected to ensure there were no linearly dependent parameters of the fit. If any variables were linearly dependent (e.g. correlation coefficient >0.99), one variable was set to be ‘0’ and was removed from the fitting procedure. All three spectra showed linear dependencies between the g strain parameters and the linewidth parameter; setting the zz (∥) value to 0 helped to lower their interdependence. An additional linear dependence was encountered between the A_{xx} and A_{yy} values for the selenide complex. Best fit parameters are listed in Table S7. The determination of these best-fit parameters would be assisted by collection of CW spectra at multiple bands; however, due to the the low g values of these compounds, they do not fall within the range of our Q-band instrument.

Table S8: Inversion recovery experimental parameters

		T / μs	T Step Size / μs	Number of Points	Shot Repetition Time / ms	Pulse Attenuation / dB
Oxide	5 K	19	19	1024	20	7
	10 K	4	4	1024	4.1	7
	15 K	1	1	1024	1.1	7
	20 K	1	1	1024	1.1	7
	25 K	1	1	1024	1.1	7
	30 K	0.5	0.5	1024	1.1	7
	40 K	0.5	0.5	1024	1	7
	50 K	0.5	0.5	1024	1	7
Sulfide	5 K	13	13	1024	13.5	12
	10 K	10	10	1024	10.5	12
	15 K	1	1	1024	1.1	12
	20 K	1	1	1024	1	13
	30 K	0.5	0.5	1024	1	12
Selenide	5 K	11	11	1024	11.5	12
	10 K	1	1	1024	1.1	12
	15 K	1	1	1024	1.1	12
	20 K	0.5	0.5	1024	1.1	12
	25 K	0.5	0.5	1024	1	12

S3.2 Hahn Spin-Echo EPR Experiments

Pulsed EPR measurements were performed using an overcoupled Bruker ER4118X-MD5 resonator with a 1 kW TWT amplifier. The field-swept echo-detected spectrum was collected for each sample at 20 K using a $(\pi/2)$–τ–π–τ pulse sequence. The attenuation of the pulses was adjusted between 7–11 dB to give the maximum echo intensity.

To collect the T_m relaxation times, the Hahn echo decay was collected using a $(\pi/2)$–τ–π–τ–echo sequence. The pulse lengths were 30 ns and 60 ns. The time τ was increased by 8 ns for 1024 points from an initial value of 400 ns. The attenuation of the pulses was adjusted between 12–13 dB to give the maximum echo intensity.

To collect the T_1 relaxation times, a three-pulse inversion recovery electron spin-echo experiment was performed at X-band at temperatures from 5 to 30 K. A π–T–$(\pi/2)$–τ–π–τ–echo sequence with pulse lengths of 60, 30, and 60 ns was used. The time τ was constant at 400 ns. Initial values of T varied based on temperature and shot repetition time. Specific parameters are provided in Table S8. Data were analyzed using an exponential decay for inversion recovery experiments or a stretched exponential decay function for the Hahn echo decay experiments. Data points are shown in Table S9 and plotted in Figure S8.

S3.3 Two-Dimensional Field-Swept Electron Spin-Echo Experiments

In this experiment, the Hahn echo decay was collected across the field-swept echo-detected spectrum. A $(\pi/2)$–τ–π–τ–echo sequence with pulse lengths of 30 and 60 ns was used. Pulse attenuation was set to 11 dB to give maximum echo intensity. The time τ was initially set to 200 ns and increased by 4 ns for a total number of points that varied from 100 to 400 depending on temperature. In the direct dimension, the data was collected over a width of 400 G with a collection at every 1 G. A two-step phase cycling procedure was used. The number of points and total collection time varied for each temperature. All details are provided in Table S10. The data was baseline-corrected and Fourier transformed in the indirect dimension. For the plots shown in Figure 3(c), the data was normalized as described before.[26,27] Larger reproductions of the plots in Figure 3(c) are shown in Figure S9 along with a plot of the data acquired at 25 K.

Table S9: Spin T_1 and T_m relaxation times

		$g_\perp$ Region			$g_\parallel$ Region		
		T_1 / ns	T_m / ns	k^a	T_1 / ns	T_m / ns	k^a
Oxide[b]	5 K	$2.821(8) \times 10^6$	1667(3)	1.334(3)	$5.311(21) \times 10^6$	1386(6)	1.463(12)
	10 K	$5.037(4) \times 10^5$	1590.0(1.4)	1.376(2)	$3.680(10) \times 10^5$	1391(4)	1.500(9)
	15 K	$7.282(10) \times 10^4$	1283.9(1.4)	1.304(2)	$6.98(3) \times 10^4$	1188(6)	1.481(14)
	20 K	$3.025(3) \times 10^4$	968(2)	1.172(4)	$2.80(3) \times 10^4$	1061(7)	1.53(2)
	25 K	$1.1294(17) \times 10^4$			$1.13(2) \times 10^4$		
	30 K	$4.838(14) \times 10^3$	750(5)	1.105(9)	$4.39(10) \times 10^3$	1023(9)	1.58(3)
	40 K	$1.83(3) \times 10^3$			$2.30(11) \times 10^3$		
	50 K	$1.23(7) \times 10^3$			$1.2(3) \times 10^3$		
Sulfide[c]	5 K	$2.888(9) \times 10^6$	1628.9(1.1)	1.1222(10)	$3.410(2) \times 10^6$	1320(4)	1.132(5)
	10 K	$1.976(4) \times 10^5$	1317.4(7)	1.1368(8)	$1.426(10) \times 10^5$	1123(3)	1.154(4)
	15 K	$2.293(4) \times 10^4$	860.4(8)	1.088(1)	$1.879(5) \times 10^4$	814(4)	1.100(7)
	20 K	$4.878(13) \times 10^3$	559(2)	1.000(4)	$4.39(8) \times 10^3$	696(7)	1.081(13)
	30 K	$9.4(3) \times 10^2$	852(9)	1.28(2)	$1.03(12) \times 10^3$	988(10)	1.55(3)
Selenide[d]	5 K	$2.256(8) \times 10^6$	1736.4(1.4)	1.1425(14)	$2.585(13) \times 10^6$	1425(3)	1.085(3)
	10 K	$9.094(13) \times 10^4$	1277.5(6)	1.1431(8)	$7.300(13) \times 10^4$	1100(2)	1.136(3)
	15 K	$1.025(2) \times 10^4$	724.4(1.0)	1.0501(19)	$9.18(3) \times 10^3$	656(3)	1.035(5)
	20 K	$2.82(3) \times 10^3$	407(2)	0.989(6)	$2.63(7) \times 10^3$	353(7)	1.03(2)
	25 K	$1.17(6) \times 10^3$	251(5)	1.06(3)	$1.3(2) \times 10^3$	197(13)	1.21(12)

[a] Stretch exponent. [b] Oxide $g_\perp$ measurements were performed at 3844 G and $g_\parallel$ at 4029 G. [c] Sulfide $g_\perp$ measurements were performed at 4128 G and $g_\parallel$ at 4264 G. [d] Selenide $g_\perp$ measurements were performed at 4216 G and $g_\parallel$ at 4332 G.

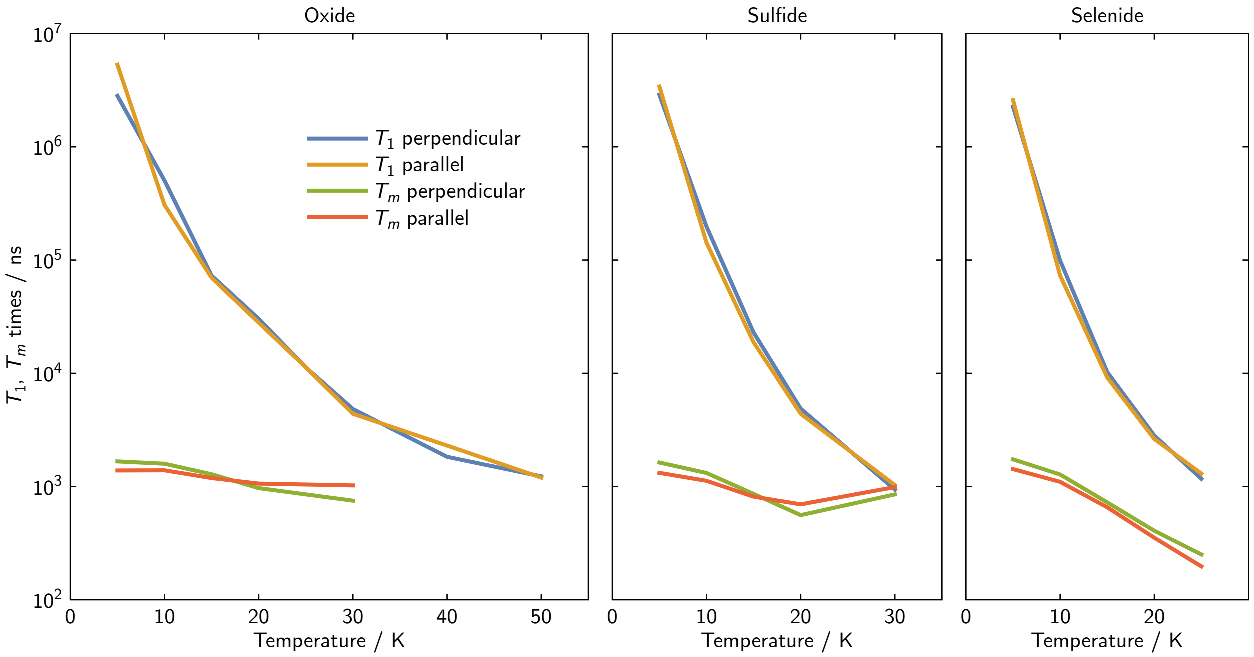

Figure S8: T_1 and T_m times obtained from inversion recovery and Hahn-echo experiments for $[Na(THF)_6][\mathbf{1}\cdot E]$ compounds dissolved in 1:1 THF/toluene.

Table S10: 2D FS-ESE experimental parameters

	Number of Points per Field	Magnetic Field Step Size / G	Number of Points in Time Domain	Time Domain Step Size / ns	Total Collection Time / ns
10 K	400	1	400	4	1600
15 K	400	1	256	4	1024
20 K	400	1	256	4	1024
25 K	400	1	125	4	500
30 K	400	1	100	4	400

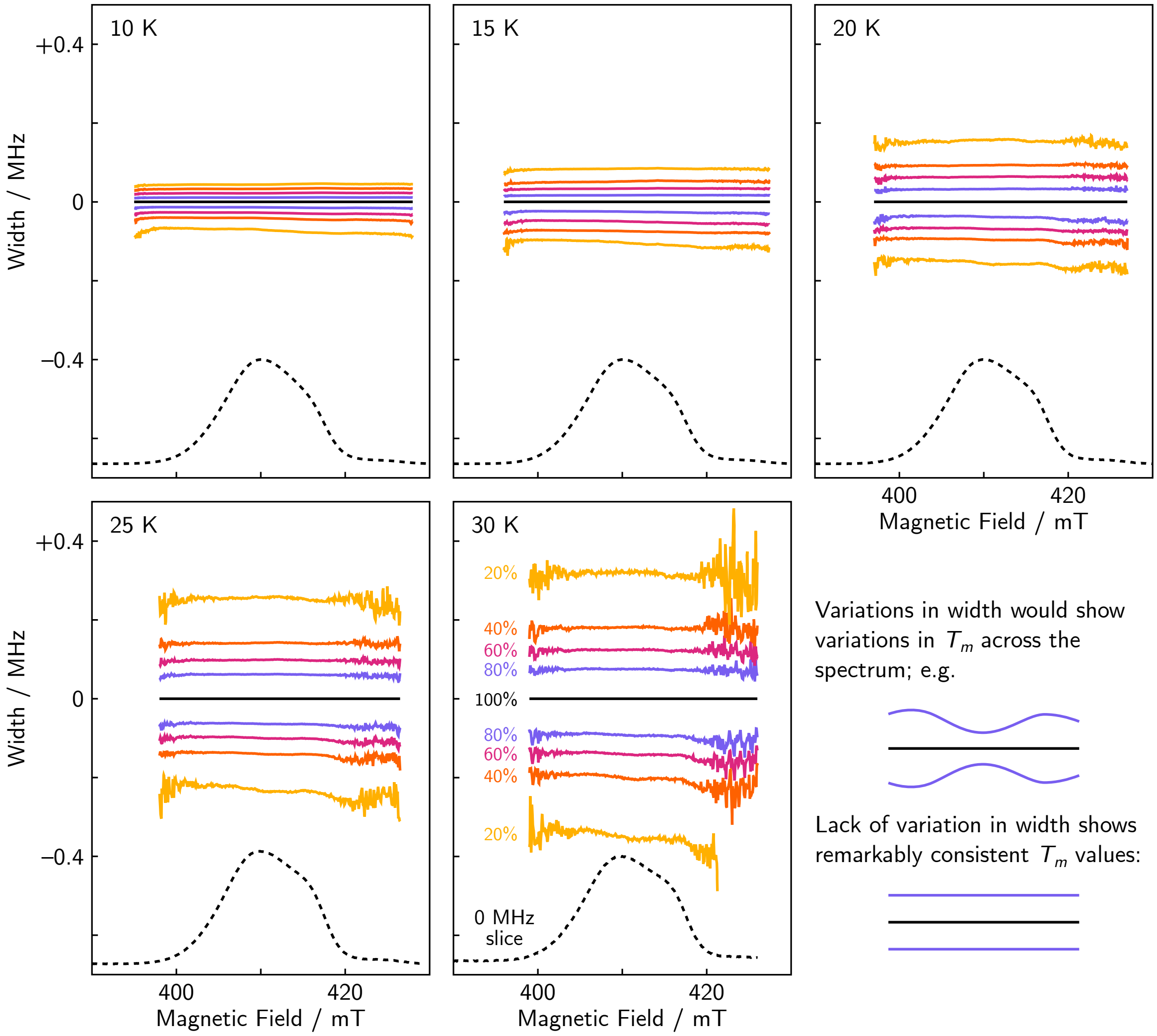

Figure S9: 2D FS-ESE normalized contour plots for a frozen 1:1 THF/toluene solution of $[Na(THF)_x(Et_2O)_y][\mathbf{1}{\cdot}Se]$ acquired at a series of temperatures. The 30 K plot has a legend for contour intensities. The dashed line is the 0 MHz trace, roughly equivalent to an EDFS (echo-detected field swept) spectrum.

S4 X-ray Crystallography

The crystals were mounted in hydrocarbon oil on a nylon loop or a glass fiber. Low-temperature (100–170 K) data were collected on a Bruker D8 Venture Duo diffractometer coupled to a Photon III CPAD with Mo Kα radiaton (λ = 0.71073 Å) at low temperature (100–170 K) with ϕ- and ω-scans. Data were indexed with Apex II (Difference Vectors method), integrated with Bruker SAINT, and a semi-empirical absorption correction was applied using SADABS or TWINABS. The space group was established using XPREP, and the structure was solved by intrinsic phasing methods using SHELXT-2018/2 and refined against F^2 on all data by full-matrix least squares with SHELXL-2019/2 using established methods. All non-hydrogen atoms were refined anisotropically. All hydrogen atoms were included in the model at geometrically calculated positions and refined using a riding model unless otherwise noted. The isotropic displacement parameters of all hydrogen atoms were fixed to 1.2 times the U_{eq} value of the atoms they are linked to (1.5 times for methyl groups).

Compound $[Na(THF)_6][\mathbf{1}\cdot O]$ crystallized in the monoclinic space group $P2_1/c$ with one molecule in the asymmetric unit. The β angle of the unit cell is close to 90°, so we attempted to solve the structure in orthorhombic space groups, but we invariably obtained inferior CFOM metrics. We settled on the monoclinic $P2_1/c$ solution, and it can be seen in Table S11 that $\beta = 90.0320(10)°$ is more than five standard uncertainties away from 90°. The ODipp ligands on the tungsten and the THF ligands on the sodium were all disordered over two positions with the help of similarity restraints on 1–2 and 1–3 distances and displacement parameters as well as rigid bond restraints for anisotropic displacement parameters. The disorder ratio for the ODipp ligands freely refined to 0.7099(29) and the disorder ratio for the THF ligands freely refined to 0.7100(35). These two free variables are equal within one standard uncertainty, so these two minor positions for the molecule likely occur together. The τ_5 parameter about the tungsten center was calculated to be 0.052(6) for the major component of the ODipp ligands and 0.057(12) for the minor component. The data were not of high quality, but we judged them to be sufficient for calculation of a τ_5 parameter to two significant figures. Only the τ_5 parameter for the major component is listed in the main manuscript.

Compound $[Na(THF)_6][\mathbf{1}\cdot S]$ crystallized in the triclinic space group $P\bar{1}$ with one molecule, one THF, and one Et_2O in the asymmetric unit. The crystal was twinned, and data anomalies were obviously present, so we do not rely heavily on any bond metrics from the structure. One ODipp ligand showed a disordered isopropyl group, and several THF ligands on the sodium ion showed disorder. The ODipp isopropyl moiety and the THF ligands on the sodium were all disordered over two positions with the help of similarity restraints on 1–2 and 1–3 distances and displacement parameters as well as rigid bond restraints for anisotropic displacement parameters. The isopropyl disorder was freely refined to a ratio of 0.7084(94), and the disorder models for the THF ligands freely refined to ratios of 0.524(21), 0.7779(69), 0.7703(82), and 0.311(29). The τ_5 parameter about the tungsten center was calculated to be 0.038(2), indicating a structure close to a square pyramid; however, these data were of low quality and this limits the quantitative reliability of the metrics for this structure.

Compound $[Na(THF)_6][\mathbf{1}\cdot Se]$ crystallized in the monoclinic space group $P2_1/c$ with one $[Na(THF)_6]^+$ cation, one $[\mathbf{1}\cdot Se]^-$ anion, and one disordered THF molecule in the asymmetric unit. Four of the THF ligands on the sodium were disordered over two positions with the help of similarity restraints on 1–2 and 1–3 distances and displacement parameters as well as rigid bond restraints for anisotropic displacement parameters. The disorder ratios for the THF ligands freely refined to 0.449(28), 0.682(12), 0.701(16), and 0.705(12). The τ_5 parameter about the tungsten center was calculated to be 0.0238(24), indicating a structure close to a square pyramid.

Figures S10–S12 were made in PLATON and show the asymmetric unit of each model.

Table S11: Summary of crystallographic data

	$[Na(THF)_6][\mathbf{1}{\cdot}O]$	$[Na(THF)_6][\mathbf{1}{\cdot}S]{\cdot}THF{\cdot}Et_2O$	$[Na(THF)_6][\mathbf{1}{\cdot}Se]{\cdot}THF$
CCDC	CSD 2428564	CSD 2428566	CSD 2428565
Empirical formula	$C_{72}H_{116}NaO_{11}W$	$C_{76}H_{125}NaO_{11}SW$	$C_{76}H_{124}NaO_{11}SeW$
Formula weight (g/mol)	1364.48	1453.65	1499.54
Color / Morphology	blue / block	teal / needle	green / block
Crystal size (mm^3)	$0.20 \times 0.17 \times 0.15$	$0.10 \times 0.25 \times 0.25$	$0.30 \times 0.25 \times 0.20$
Temperature (K)	170(2)	100(2)	150(2)
Wavelength (Å)	0.71073	0.71073	0.71073
Crystal system, Space group	Monoclinic, $P2_1/c$	Triclinic, $P\bar{1}$	Monoclinic, $P2_1/c$
Unit cell dimensions (Å, °)	$a = 14.1273(4)$, $\alpha = 90$	$a = 13.8766(7)$, $\alpha = 87.403(2)$	$a = 13.9588(5)$, $\alpha = 90$
	$b = 18.6952(6)$, $\beta = 90.0320(10)$	$b = 14.5147(8)$, $\beta = 83.184(2)$	$b = 14.6100(5)$, $\beta = 100.2640(10)$
	$c = 27.5367(7)$, $\gamma = 90$	$c = 19.2305(10)$, $\gamma = 89.002(2)$	$c = 38.6797(13)$, $\gamma = 90$
Volume (Å^3)	7272.8(4)	3841.7(5)	7762.0(5)
Z	4	2	4
Density (calc., g/cm^3)	1.246	1.257	1.283
Absorption coefficient (mm^{-1})	1.649	1.591	2.017
$F(000)$	2884	1540	3148
Theta range for data collection (°)	2.335 to 27.581	2.027 to 33.855	2.035 to 30.516
Index ranges	$-18 \le h \le 18$, $-24 \le k \le 24$,	$-21 \le h \le 21$, $-22 \le k \le 22$,	$-19 \le h \le 19$, $-20 \le k \le 20$,
	$-35 \le l \le 35$	$-30 \le l \le 30$	$-45 \le l \le 55$
Reflections collected	369515	278948	422049
Independent reflections, R_{int}	16752, 0.0791	30712, 0.0894	23655, 0.0819
Completeness to $\theta_{\max}$ (%)	99.5	99.7	99.9
Absorption correction	Multi-Scan	Multi-Scan	Multi-Scan
Refinement method	Full-matrix least squares on F^2	Full-matrix least squares on F^2	Full-matrix least squares on F^2
Data / Restraints / Parameters	16752 / 5560 / 1538	30712 / 2801 / 1088	23655 / 2718 / 975
Goodness-of-fit[a]	1.283	1.216	1.368
Final R indices[b] $[I > 2\sigma(I)]$	$R_1 = 0.0534$, $wR_2 = 0.1391$	$R_1 = 0.0424$, $wR_2 = 0.1001$	$R_1 = 0.0515$, $wR_2 = 0.1140$
R indices[b] (all data)	$R_1 = 0.0649$, $wR_2 = 0.1433$	$R_1 = 0.0519$, $wR_2 = 0.1036$	$R_1 = 0.0556$, $wR_2 = 0.1152$
Largest diff. peak and hole ($e{\cdot}Å^{-3}$)	1.209 and −1.713	2.706 and −4.325	1.935 and −3.722

[a] Goodness-of-fit $= \sqrt{\frac{\sum[w(F_o^2-F_c^2)^2]}{(n-p)}}$ [b] $R_1 = \frac{\sum||F_o|-|F_c||}{\sum|F_o|}$; $wR_2 = \sqrt{\frac{\sum[w(F_o^2-F_c^2)^2]}{\sum[w(F_o^2)^2]}}$; $w = \frac{1}{\sigma^2(F_o^2)+(aP)^2+bP}$; $P = \frac{2F_c^2+\max(F_o^2,0)}{3}$

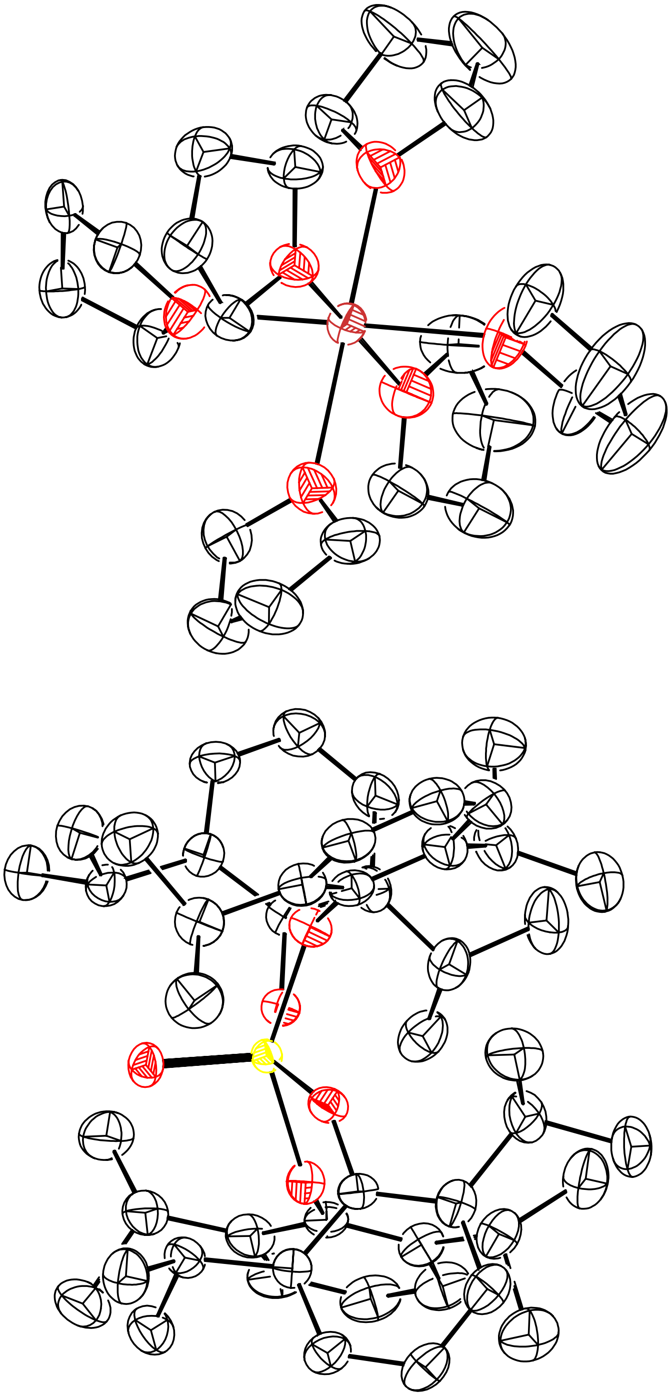

Figure S10: Thermal ellipsoid (50%) plot for the asymmetric unit of the crystal structure of [Na(THF)$_6$][**1**·O]. Disorder and hydrogen atoms were omitted for clarity.

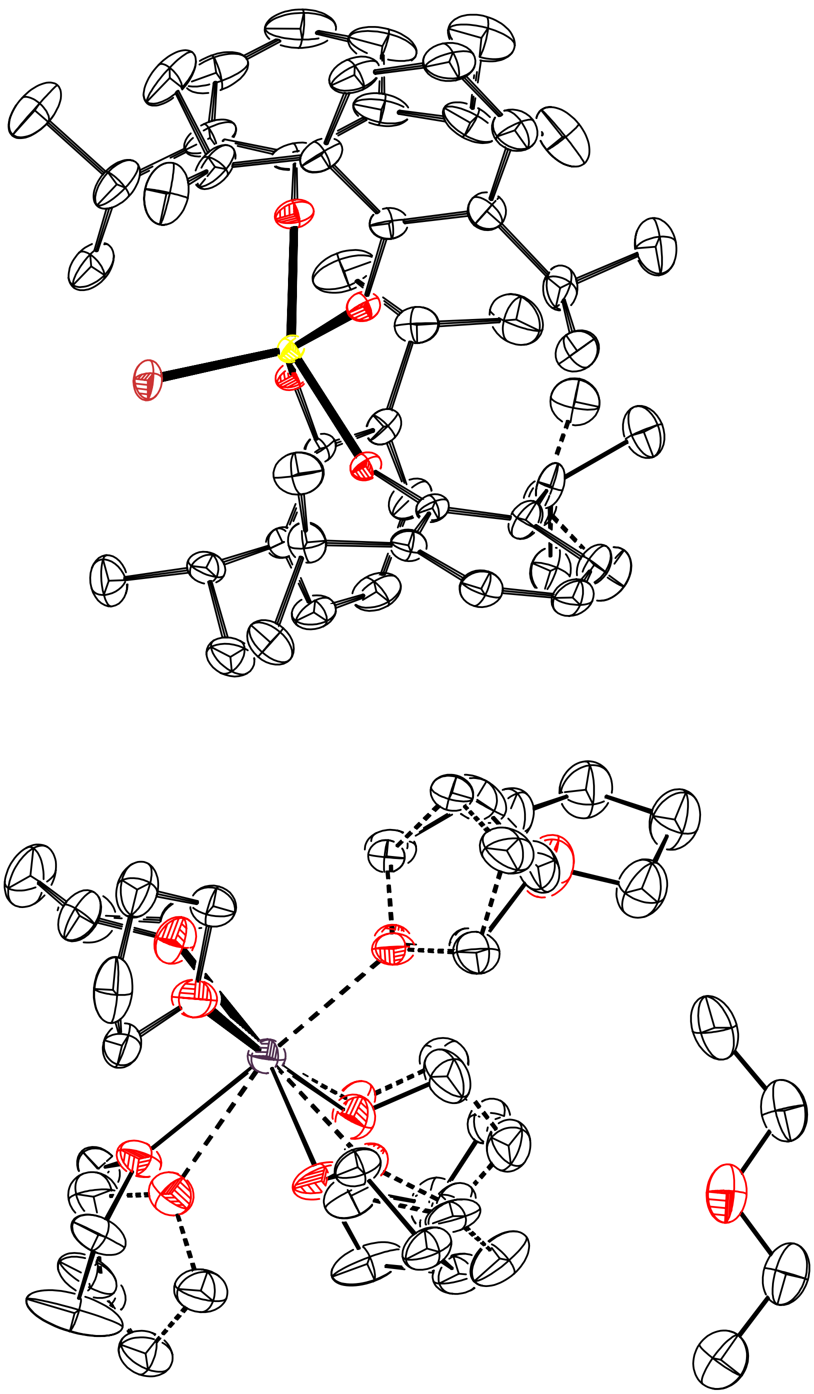

Figure S11: Thermal ellipsoid (50%) plot for the asymmetric unit of the crystal structure of [Na(THF)$_6$][**1**·S]. Hydrogen atoms were omitted for clarity.

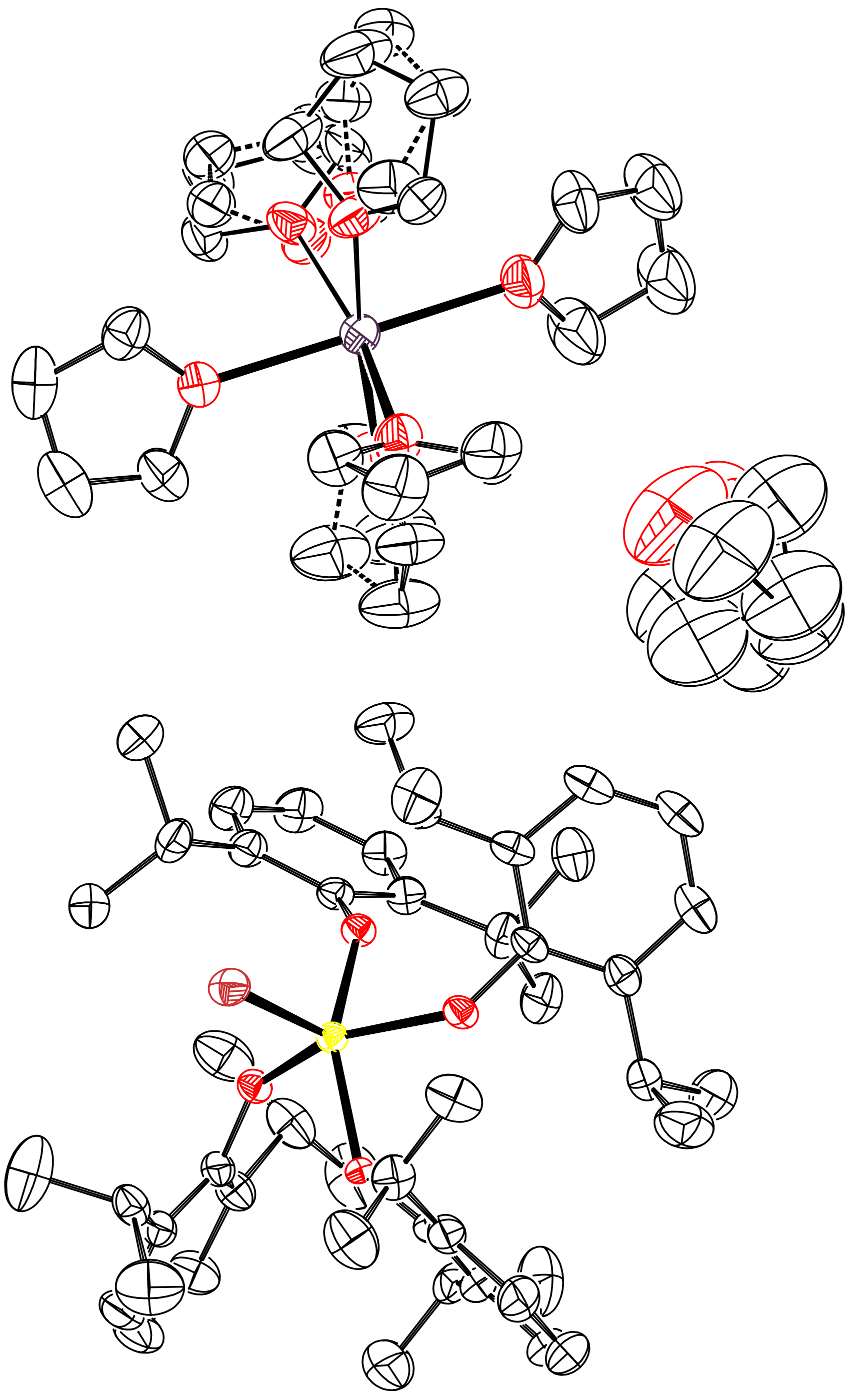

Figure S12: Thermal ellipsoid (50%) plot for the asymmetric unit of the crystal structure of [Na(THF)$_6$][**1**·Se]. Hydrogen atoms were omitted for clarity.

S5 Computational Methods

All calculations were performed using Orca 6.0.1.[28–37]

S5.1 CASSCF/RI-NEVPT2 Multireference Calculations

Initial XYZ coordinates for each anion of interest were taken from refined crystallographic CIF files. The coordinates were translated and rotated so that the tungsten atoms lied at the origin, the tungsten–chalcogenide bond was aligned along the z axis, and one tungsten–oxygen$_{\mathrm{ODipp}}$ bonds lied in the xz plane. Each geometry was optimized using the B3LYP density functional,[38,39] the ma-Def2-SVP basis set on all non-tungsten atoms,[40,41] the Def2-ECP effective core potential on the tungsten atoms,[42] the D3(BJ) dispersion correction,[43,44] and a conductor-like polarizable continuum model to describe tetrahydrofuran solvation.[45] The calculations used the default RIJCOSX approximation[46–48] and the Def2/J auxiliary basis[49] set on all atoms. The input file was

```
! D3BJ B3LYP ma-Def2-SVP Def2/J Opt CPCM(THF)
%maxcore 7000
%pal nprocs 16 end
*xyzfile -1 2 anion-from-xray-structure.xyz
```

The optimized geometries for the anions are listed below (Sec. S5.2).

Multireference calculations are more computationally expensive than density functional theory ones, so a truncated series of model complexes $[\mathrm{WE(OPh)_4}]^-$ was constructed. For each anion, the DFT-optimized structure was modified by truncating the isopropyl groups into hydrogen atoms using Avogadro, and the hydrogen atom positions were then reoptimized. Because the steric influence of isopropyl groups likely has a strong influence on the symmetry and relative alignment of the aryloxide ligands, the non-hydrogen atoms were not allowed to vary during this hydrogen atom reoptimization. The ORCA input file was

```
! D3BJ B3LYP ma-Def2-SVP Def2/J Opt CPCM(THF)
%maxcore 7000
%pal nprocs 16 end
%geom
  OptimizeHydrogens true  # only let H atoms move
  end
*xyzfile -1 2 init.xyz
```

The optimized geometries for the model complexes are listed below (Sec. S5.3).

S5.1.1 A Minimal (1,5) Active Space

Multireference calculations require careful selection of orbitals for inclusion in the active space, and the results can vary strongly depending on which orbitals are chosen to be included. We ran our calculations with multiple sizes of active spaces, beginning with a minimal (1,5) active space using one electron among the five d orbitals. In preparation for multireference calculations, quasi-restricted orbitals were obtained using

```
! DKH D3BJ B3LYP DKH-def2-SVP SARC/J UNO
%maxcore 7000
%pal nprocs 16 end
%basis
  NewGTO W "SARC-DKH-TZVP" end
  NewGTO O "DKH-def2-TZVP" end
  NewGTO S "DKH-def2-TZVP" end
  NewGTO Se "DKH-def2-TZVP" end
  end
*xyzfile -1 2 optimized.xyz
```

As seen in this input file, all of these calculations used a DKH relativistic correction and relativistically-contracted basis sets: the tungsten used the SARC-DKH-TZVP basis set, all chalcogens (including those on the OPh^- ligands) used the DKH-def2-TZVP basis set, and all other atoms used the smaller DKH-def2-SVP basis set.[31,40,50] This mixture of basis sets was chosen to allow for more wavefunction flexibility in the proximity of the active space orbitals, and it was used for all multireference calculations below.

A state-averaged CASSCF(1,5)/RI-NEVPT2 calculation was performed using the five tungsten d orbitals in the active space and requesting all five possible $5d^1$ doublet states:

```
! DKH DKH-def2-SVP AutoAux MORead RI-NEVPT2
%moinp "rotated-orbitals.gbw"
%maxcore 7000
%pal nprocs 16 end
%basis
  NewGTO W "SARC-DKH-TZVP" end
  NewGTO O "DKH-def2-TZVP" end
  NewGTO S "DKH-def2-TZVP" end
  NewGTO Se "DKH-def2-TZVP" end
  end
%casscf
  nel 1
  norb 5
  mult 2
  nroots 5
  IntOrbs PMOs  # find bonding partners for each active orbital
  ActOrbs SDO
  rel
    dosoc true
    gtensor true
    end
  end
*xyzfile -1 2 optimized.xyz
```

The vertical excitation energies for the d–d transitions of each compound are given in the upper part of Table S12.

This minimal (1,5) active space is conceptually useful for interpretation using ligand field theory, but such small active spaces are known to lead to overly ionic metal–ligand interactions and overestimated energies of ligand field transitions—especially for metals in high oxidation states bonded to soft ligands.[51] There is a clear linear relation between the observed experimental energies and the predicted values from model complexes (after accounting for SOC), and this can be seen in Figure S13. However, it is peculiar that the CASSCF(1,5) energies appear to have better agreement with experiment before addition of the NEVPT2 correction, and we ascribe this to the small size of the active space (see below).

S5.1.2 Larger (5,7) and (9,9) Active Spaces and the Role of Covalency

Addition of more electrons and orbitals into the active space improved agreement with experiment and removed the peculiarity where the NEVPT2 correction hurt agreement with experiment. We explored two larger active spaces: a (5,7) active space and a (9,9) active space.

The (5,7) active space included the five d orbitals of the tungsten plus the two covalent $\pi_{W\equiv E}$ bonds. The five electrons comprise the one $5d^1$ electron plus the four electrons in these filled π bonds. The initial CASSCF(5,7) guess orbitals were taken from the output of the CASSCF(1,5) using the `IntOrbs PMOs` subroutine in ORCA to find bonding partners for each orbital in the active space (see above input file). The new input file read

```
! DKH DKH-def2-SVP AutoAux MORead RI-NEVPT2
%moinp "casscf-1-5.gbw"
%maxcore 7000
```

Table S12: Predicted energies[a] from CASSCF/NEVPT2 calculations on model complexes

		$[WO(OPh)_4]^-$				$[WS(OPh)_4]^-$				$[WSe(OPh)_4]^-$			
		CASSCF	NEVPT2	+SOC[b]	Exp't[c]	CASSCF	NEVPT2	+SOC[b]	Exp't[c]	CASSCF	NEVPT2	+SOC[b]	Exp't[c]
(1,5)[d]	$(xy)^1$	0	0	0	0	0	0	0	0	0	0	0	0
	$(xz)^1$	18132.1	20341.3	19060.7	14566(26)	10293.4	11955.1	10840.9	7582(28)	8236.1	10071.2	9239.5	6537(14)
	$(yz)^1$	18204.7	20403.4	21995.9	17496(61)	10717.5	12290.6	14018.2	10375(36)	10372.9	11794.7	13368.2	9525(10)
	$(z^2)^1$	30797.1	31657.5	32453.8	—	19710.0	20749.7	21683.7	16701(41)	17456.4	18514.4	19433.7	15250(15)
	$(x^2-y^2)^1$	34990.8	33944.5	34793.5	—	34529.0	33247.7	34082.3	—	33913.9	32518.1	33775.1	—
(5,7)[d]	$(xy)^1$	0	0	0	0	0	0	0	0	0	0	0	0
	$(xz)^1$	21730.6	20201.7	19163.1	14566(26)	12714.6	11171.7	10272.2	7582(28)	10587.5	9369.3	8791.8	6537(14)
	$(yz)^1$	21792.9	20263.4	21552.6	17496(61)	13030.7	11523.0	12867.3	10375(36)	12062.9	11157.2	12208.0	9525(10)
	$(z^2)^1$	29655.7	29884.7	30607.2	—	18345.5	18991.2	19841.5	16701(41)	15656.0	16622.4	17492.5	15250(15)
	$(x^2-y^2)^1$	34958.3	33608.7	34358.8	—	34301.7	34080.4	34920.7	—	33854.1	33121.8	33958.7	—
(9,9)[d]	$(xy)^1$	0	0	0	0	0	0	0	0	0	0	0	0
	$(xz)^1$	21387.1	19411.6	18480.3	14566(26)	12351.0	10274.9	9510.1	7582(28)	10390.9	8568.1	8177.2	6537(14)
	$(yz)^1$	21256.6	19522.1	20825.2	17496(61)	12728.2	10707.2	12108.1	10375(36)	12108.9	10397.0	11531.2	9525(10)
	$(z^2)^1$	35153.5	31754.0	32443.6	—	21726.7	18974.6	19728.7	16701(41)	19348.7	17036.1	17751.1	15250(15)
	$(x^2-y^2)^1$	35330.4	32402.6	33124.9	—	34745.8	33162.3	33548.1	—	33609.0	32150.3	32986.1	—

[a] All values in cm^{-1}. [b] The notion of orbitals is blurred by SOC between such closely spaced orbitals as the nearly-degenerate $\{xz, yz\}$ orbitals, so the first and second excited states do not neatly fit into $(xz)^1$ and $(yz)^1$ labels. [c] Values from lineshape fitting of THF solutions of $[\mathbf{1}\cdot\mathrm{E}]^-$ complexes. [d] These two numbers describe the CASSCF active space: the numbers of electrons first and the number of orbitals second.

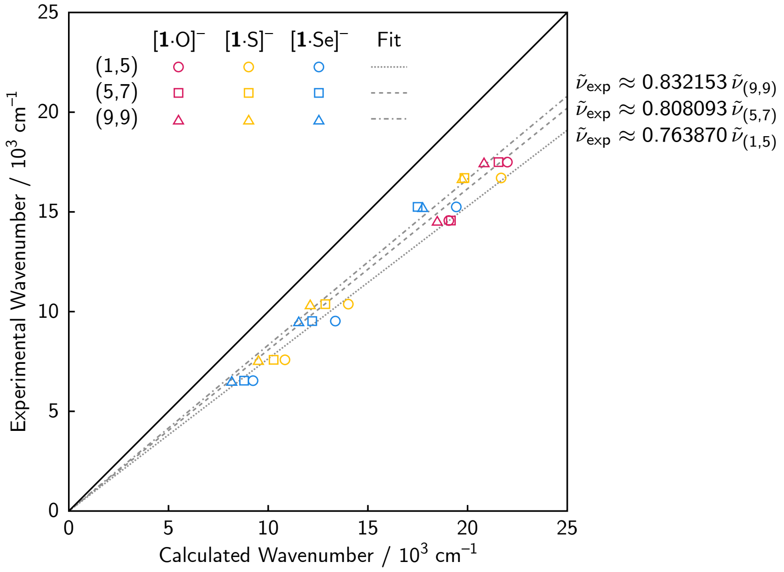

Figure S13: The calculated (CASSCF/RI-NEVPT2+SOC) wavenumbers are overestimated but they correlate closely with the experimental values through the gray linear regression. The agreement with experiment improved as we increased the size of the active space: the (5,7) active space included the $\pi_{W\equiv E}$ orbitals, and the (9,9) active space included those plus the $\sigma_{W\equiv E}$ orbital and a b_1-symmetry orbital that was the bonding partner to the $x^2{-}y^2$ d orbital.

```
%pal nprocs 16 end
%basis
  NewGTO W "SARC-DKH-TZVP" end
  NewGTO O "DKH-def2-TZVP" end
  NewGTO S "DKH-def2-TZVP" end
  NewGTO Se "DKH-def2-TZVP" end
  end
%casscf
  nel 5
  norb 7
  mult 2
  nroots 40
  rel
    dosoc true
    gtensor true
    end
  end
*xyzfile -1 2 optimized.xyz
```

These state-averaged CASSCF(5,7)/RI-NEVPT2 calculations requested the first forty doublet roots (ground state plus 39 excited states) out of the theoretically possible 735 doublet roots. For all calculations, the first four roots were the $(xy)^1$, $(xz)^1$, $(yz)^1$, and $(z^2)^1$ states, but the $(x^2{-}y^2)^1$ root often appeared at higher energies and had to be manually located. The vertical excitation energies for the d–d transitions of each compound are given in the middle part of Table S12.

A larger (9,9) active space was also explored. This active space used all electrons/orbitals of the (5,7) active space, and added the $\sigma_{W\equiv E}$ orbital (bonding counterpart to the d_{z^2} orbital) and a b_1 orbital describing the W–ODipp interactions (the bonding counterpart to the $d_{x^2-y^2}$ orbital). The initial orbitals were taken from the CASSCF(1,5) results. The state-averaged CASSCF(9,9)/RI-NEVPT2 calculation requested the first eighty doublet roots out of the theoretically possible 15,876 doublets. For all calculations, the first four

Table S13: Predicted g values from CASSCF/NEVPT2 calculations on model complexes

		$[WO(OPh)_4]^-$			$[WS(OPh)_4]^-$			$[WSe(OPh)_4]^-$		
		CASSCF	NEVPT2	Exp't[a]	CASSCF	NEVPT2	Exp't[a]	CASSCF	NEVPT2	Exp't[a]
(1,5)[b]	g_{xx}	1.762773	1.783795	1.8007	1.641250	1.679801	1.6765	1.664696	1.630344	1.6400
	g_{yy}	1.760945	1.782289	1.8007	1.616589	1.659988	1.6698	1.649751	1.536359	1.6289
	g_{zz}	1.524204	1.523413	1.7151	1.439120	1.452662	1.6208	1.461183	1.395475	1.5952
(5,7)[b]	g_{xx}	1.848881	1.834485	1.8007	1.751951	1.724432	1.6765	1.630344	1.671084	1.6400
	g_{yy}	1.847998	1.833498	1.8007	1.739286	1.708828	1.6698	1.536359	1.602178	1.6289
	g_{zz}	1.568878	1.547802	1.7151	1.515542	1.497896	1.6208	1.395475	1.417923	1.5952
(9,9)[b]	g_{xx}	1.824508	1.808256	1.8007	1.709115	1.664696	1.6765	1.674513	1.634102	1.6400
	g_{yy}	1.821504	1.801366	1.8007	1.697262	1.649751	1.6698	1.631585	1.580373	1.6289
	g_{zz}	1.579702	1.535216	1.7151	1.510881	1.461183	1.6208	1.477680	1.423208	1.5952

[a] Values from EasySpin fits of [**1**·E]$^-$ EPR spectra (see Table S7). [b] These two numbers describe the CASSCF active space: the numbers of electrons first and the number of orbitals second.

roots were the $(xy)^1$, $(xz)^1$, $(yz)^1$, and $(z^2)^1$ states, but the $(x^2{-}y^2)^1$ root often appeared at higher energies and had to be manually located. The vertical excitation energies for the $d{-}d$ transitions of each compound are given in the lower part of Table S12.

It is clear that adding in additional bonding orbitals into the active space improved the agreement of these multireference calculations with experiment. This same improvement in experimental agreement can be seen in the predicted g values for the model complexes (Table S13). This shows that covalent mixing between d orbitals and ligand-centered orbitals plays a large role in the physical properties of these systems, especially in tempering the deviation of g values from 2.0023 due to relativistic nephelauxesis.[52] The stronger deviation from experiment in the g_{zz} direction suggests the $x^2{-}y^2$ orbital still needs higher levels of covalency than the (9,9) active space captures. The increase in rhombicity for the sulfide and selenide species appears to reflect an increase in the τ_5 parameter predicted by the DFT calculations: [**1**·O]$^-$ optimized to $\tau_5 = 0.0067$, [**1**·S]$^-$ to $\tau_5 = 0.0167$, and [**1**·Se]$^-$ to $\tau_5 = 0.1183$.

S5.2 Optimized $[WE(ODipp)_4]^-$ XYZ Coordinates

Tungsten Oxide

W	0.02038687267774	-0.01004442297235	-0.17867538252649
O	0.02214470789762	0.00011197494679	1.54903207451237
O	1.92112995208009	-0.03346717323936	-0.75034693779833
C	2.87660354562941	0.85837376027883	-1.07393894932105
C	3.39780949494537	1.76273553683370	-0.11672693918015
C	2.89539028429574	1.78903930829056	1.31752460832622
H	2.20062502133044	0.95188105165298	1.44328426666832
C	2.11739708005379	3.08051004673017	1.61659637222590
H	2.77459639852818	3.96387959139928	1.55078346328450
H	1.68899519589792	3.05241225217340	2.63221391628000
H	1.29403360231994	3.20761377522749	0.90165363709135
C	4.03255131628297	1.57795325659127	2.32956223136149
H	4.75260578567331	2.41282739321489	2.31189456680676
H	4.58402037053258	0.64805783058872	2.12008524653273
H	3.62657799751478	1.51037892784336	3.35268752908934
C	4.41469068056194	2.64300184279318	-0.51427990490007
H	4.81543778682505	3.35363091266038	0.21337634230487
C	4.93264679845254	2.62462052589966	-1.80946038715913
H	5.72780358815791	3.31750308800207	-2.09703858650893
C	4.42815995084999	1.70586875433325	-2.73401416560992
H	4.84042621756846	1.68567860220474	-3.74452510479086
C	3.40091747026924	0.82159175985952	-2.39206166420622
C	2.89763834925858	-0.22682979393208	-3.36962440932199
H	1.84193046082865	-0.42270326121927	-3.12496816733398
C	3.67057618576615	-1.54035184899237	-3.15633943460281
H	4.74573922539441	-1.38281381822331	-3.34612412069413
H	3.31104078816072	-2.32616909445846	-3.83699307081258
H	3.54602951985531	-1.91053994846049	-2.12979865762428
C	2.96776542297019	0.21041801029228	-4.83693453001063
H	4.00890917958543	0.30561967061421	-5.18607977065670
H	2.46714291760584	1.17633871445429	-4.99753163748639
H	2.47750421467635	-0.53689476354526	-5.47990290669315
O	0.04686999697840	1.88798976356730	-0.75329228571307
C	-0.83254891423032	2.84673732922571	-1.09787068002751
C	-1.74943057938726	3.37853470999021	-0.15824427685548
C	-1.80338858280010	2.88119861209726	1.27713392310255
H	-0.97823590414946	2.17457862962040	1.41662860544842
C	-3.10989856897894	2.12321623760964	1.56164194218688
H	-3.98318374901369	2.79170128962163	1.47764325002500
H	-3.10269264979410	1.70257418513427	2.58079394250124
H	-3.23697640472091	1.29578483212237	0.85139708921307
C	-1.58943429778206	4.01891071620434	2.28793711288111
H	-2.41292496271889	4.75149152053899	2.25516942946441
H	-0.64828537418716	4.55532173270985	2.08992954979045
H	-1.54299348384219	3.61617859754474	3.31352010750095
C	-2.61525705131809	4.39943109874617	-0.57530419542366
H	-3.33549109784849	4.80873633425427	0.13807782317886
C	-2.57120489623338	4.91099492145469	-1.87289641877383
H	-3.25287439467312	5.71022467520496	-2.17557886506625
C	-1.64082316760485	4.39537459216489	-2.77910353390640
H	-1.59922165784500	4.80243175409302	-3.79149255758315

C	-0.77046323748475	3.36259066787396	-2.41770303499991
C	0.28540664469554	2.84721989463467	-3.38141103480324
H	0.54862610111224	1.82735897032447	-3.06445641985275
C	1.55626260556168	3.70580122836259	-3.26130518356829
H	1.33688081222171	4.75282089690034	-3.53071436881033
H	2.34849635459296	3.33472851521511	-3.92828875849980
H	1.95227897879066	3.68585820576506	-2.23589409204029
C	-0.19323575764032	2.77816804490891	-4.83627737938955
H	-0.38985473980113	3.77939078712932	-5.25299714875223
H	-1.11521977991469	2.18619207458928	-4.92742482876299
H	0.57582845093811	2.30818843390771	-5.46871702673158
O	-1.88145806768864	0.00791351237030	-0.74725455388657
C	-2.83818741693938	-0.88700631072838	-1.05842752903075
C	-3.35808312572377	-1.78007955920303	-0.08982347308682
C	-2.85277055691984	-1.79074137974803	1.34363158844297
H	-2.15802390009786	-0.95208979826639	1.45893942487869
C	-2.07372854728601	-3.07879892364384	1.65453741961051
H	-2.73127594070205	-3.96274595890255	1.60113448531546
H	-1.64166312795163	-3.03918442904857	2.66820235228479
H	-1.25307182178801	-3.21394000823919	0.93806528267513
C	-3.98779597573682	-1.56970817226172	2.35594432271099
H	-4.70736079086660	-2.40512732117012	2.34833300994626
H	-4.54034384635086	-0.64232610812237	2.13834048184394
H	-3.57952597227892	-1.49152793215604	3.37739243099124
C	-4.37606275110467	-2.66442090171422	-0.47529249262195
H	-4.77552450802908	-3.36648050472003	0.26132693174508
C	-4.89653779843144	-2.66080030648283	-1.76959705009990
H	-5.69258781537687	-3.35656376193112	-2.04759903795825
C	-4.39340914066079	-1.75299738721754	-2.70560156623400
H	-4.80759320478361	-1.74417952961764	-3.71550377713150
C	-3.36514671785144	-0.86526850370418	-2.37583875082037
C	-2.86401734711926	0.17140150063246	-3.36696307486253
H	-1.81031052482700	0.37652375460278	-3.12164228016551
C	-3.64425221807980	1.48397608083527	-3.17627317803427
H	-4.71808038805372	1.31832127027607	-3.36679828932406
H	-3.28645819495414	2.26090592692360	-3.86804915516208
H	-3.52498653604579	1.87096256747656	-2.15524584930588
C	-2.92620740678252	-0.28859230670403	-4.82770028777805
H	-3.96529387102865	-0.39762622437874	-5.17885352832798
H	-2.41712303631826	-1.25269000695938	-4.97112523142469
H	-2.43969653459804	0.45270319899591	-5.48041182077130
O	-0.00768011414312	-1.91471440275155	-0.73128027404886
C	0.86941607236362	-2.87969762785294	-1.06431847001435
C	1.78708373669917	-3.40059679344305	-0.11923409451302
C	1.84453986595947	-2.88453772948651	1.30941555060630
H	1.02015297242178	-2.17569312049684	1.44152091550102
C	3.15213386965053	-2.12394317656181	1.58153004146446
H	4.02467650791329	-2.79425461170403	1.50462919605297
H	3.14721765521309	-1.69021329803485	2.59521826193679
H	3.27897883970256	-1.30562057795480	0.86074648812641
C	1.63226421921715	-4.00895721167705	2.33542627119496
H	2.45594021811958	-4.74162153827692	2.31087619407013
H	0.69094992420272	-4.54805462699552	2.14582520945203
H	1.58736600046484	-3.59280387094559	3.35569550728391

C	2.65060691965529	-4.42829171155499	-0.52419340895339
H	3.37140971498780	-4.82921473442912	0.19333858577223
C	2.60344728088198	-4.95712609642916	-1.81472366717320
H	3.28325842786094	-5.76148820425780	-2.10784427085661
C	1.67224274170128	-4.45216310851329	-2.72602724597118
H	1.62810391400477	-4.87269542788585	-3.73277905756787
C	0.80433223640386	-3.41307560167438	-2.37705161956403
C	-0.25144666957198	-2.90858931555592	-3.34661368233537
H	-0.51435456083261	-1.88506881367539	-3.04142932989351
C	-1.52258812639838	-3.76528198379448	-3.21672631177207
H	-1.30355042935439	-4.81555129753388	-3.47347932022907
H	-2.31450241796306	-3.40213846679042	-3.88849897598283
H	-1.91905133560075	-3.73297067380106	-2.19181850956827
C	0.22765044510082	-2.85626076460624	-4.80213535270982
H	0.42472785482052	-3.86213456926442	-5.20725387538496
H	1.14941328026585	-2.26497898620602	-4.89962449150962
H	-0.54125903474017	-2.39376584839732	-5.44023728081028

Tungsten Sulfide

W	0.01998860346528	-0.00929619858735	-0.08441115760178
S	0.02137217412708	0.00396178257945	2.10467638197330
O	1.91672020595228	0.02989786553836	-0.65058126702123
C	2.86799608588868	0.90748083418301	-1.02991848237719
C	3.37601321031336	1.88932426061993	-0.14245426850091
C	2.86403816339257	2.03331219032015	1.27803634254369
H	2.12706855583365	1.24440150777294	1.44464030878936
C	2.14326823529220	3.37222599297859	1.49537458717815
H	2.83240873928576	4.22568798366598	1.37883451626728
H	1.71485974780881	3.42032689494530	2.51016850184315
H	1.32109823772415	3.48912506039583	0.77577345957203
C	3.97751473120172	1.81668274302507	2.31428655465005
H	4.75012411012058	2.60162733789638	2.25565450368688
H	4.46943336954631	0.84201705796864	2.16669229827836
H	3.55902681735693	1.83521626516963	3.33464448906777
C	4.40554953364951	2.73066658499168	-0.58807734696383
H	4.79410880077574	3.49434451579479	0.09074951534713
C	4.95162727051006	2.60615824612028	-1.86428494219249
H	5.75806674431792	3.26829333787960	-2.19010150268305
C	4.45668951228658	1.62068026074161	-2.72121313317173
H	4.88761446723501	1.51907287199108	-3.71859536768532
C	3.41664648239319	0.77077120755594	-2.33256912266675
C	2.92975835485362	-0.33615770406783	-3.25107391825417
H	1.87138171409518	-0.52308154012935	-3.01128550964674
C	3.70484471028058	-1.63138985937106	-2.95655407494486
H	4.78181906321224	-1.47928688536639	-3.14018115010386
H	3.35568441060279	-2.45249552981205	-3.59910831350330
H	3.56737976741118	-1.94793927307588	-1.91439444098261
C	3.02692307012918	0.02049373792173	-4.73913006183521
H	4.07468634047030	0.09417395928877	-5.07251102732412
H	2.53325069726117	0.97768194560476	-4.96194447827253
H	2.54921857849726	-0.75955188345942	-5.35100865143019
O	0.00167224334586	1.87210674112519	-0.67587766123783
C	-0.84200108722441	2.83798120790754	-1.08809914087150
C	-1.78912822584577	3.41718675480386	-0.20746205810325

C	-1.93236667171540	2.95115193923432	1.22995385491457
H	-1.12706137101105	2.23730964450507	1.42559606126763
C	-3.25766839757194	2.20641819514538	1.45439317433299
H	-4.12294104723434	2.87213580406364	1.29528177597117
H	-3.31396601172942	1.81763864621736	2.48463711669350
H	-3.34243029475725	1.35495648464080	0.76527337897630
C	-1.75605061400840	4.10020133446982	2.23374612263817
H	-2.56527123422449	4.84521657265501	2.15287227000669
H	-0.79751309266242	4.61959883973661	2.07631150250811
H	-1.76574987120251	3.70982517114854	3.26516860876631
C	-2.59958234842967	4.45629064057685	-0.68514433999501
H	-3.34051948746330	4.89940868193842	-0.01473798815194
C	-2.47371214683415	4.94247307687299	-1.98643139663212
H	-3.11140095634082	5.75803432517952	-2.33733642531438
C	-1.51836423610587	4.37793419948496	-2.83458362367025
H	-1.41187640688966	4.76363902894161	-3.85061424096894
C	-0.70089209623991	3.32428499922322	-2.41325778314530
C	0.37699449898641	2.76494321510794	-3.32605451756970
H	0.63974661375686	1.76491854615970	-2.95255814338409
C	1.63835971516696	3.63893463558949	-3.23168161784166
H	1.41399235701369	4.67012986519407	-3.55258217944968
H	2.43997654269832	3.24247892007332	-3.87180881340090
H	2.02229067459999	3.66960331364247	-2.20216913213059
C	-0.07845310433513	2.61906029635740	-4.78292588067684
H	-0.26657670246170	3.59720935195020	-5.25441740223727
H	-1.00001077468744	2.02532486005357	-4.85680928092865
H	0.69839450988870	2.11500775662613	-5.37808213961654
O	-1.87725373862346	-0.05547800383619	-0.64829464119006
C	-2.82886580241210	-0.93806176861925	-1.01513460279000
C	-3.33595227467023	-1.90837315268573	-0.11448825028221
C	-2.82103680382689	-2.03545771807975	1.30652083417149
H	-2.08337358736627	-1.24492801704623	1.46190875025867
C	-2.10013141955970	-3.37201946545036	1.53780407748922
H	-2.78999712511512	-4.22640973560686	1.43300405987736
H	-1.66908422075596	-3.40848651879211	2.55196444733776
H	-1.28004734320042	-3.49765086438486	0.81735233775980
C	-3.93203811950447	-1.80649002375734	2.34274361471647
H	-4.70477128636001	-2.59203180414131	2.29510841206079
H	-4.42428458677119	-0.83358478291531	2.18497627793443
H	-3.51114013464953	-1.81310442704788	3.36225467442539
C	-4.36680698516374	-2.75456706254659	-0.54771800121610
H	-4.75481781902061	-3.50928054789064	0.14138589332904
C	-4.91486629022183	-2.64604540988779	-1.82452401010990
H	-5.72237580952194	-3.31167090413410	-2.14043616228663
C	-4.42053613560237	-1.67203925003913	-2.69480995224428
H	-4.85295785648108	-1.58276828559270	-3.69270740845993
C	-3.37940369169740	-0.81789112016456	-2.31861345014123
C	-2.89370064486857	0.27727149375976	-3.25177440580629
H	-1.83467757738014	0.46645601426217	-3.01657650552278
C	-3.66765989515017	1.57634713290867	-2.97157310268333
H	-4.74507791332675	1.42225828440149	-3.15090947389107
H	-3.31982893632953	2.38931569887375	-3.62507594456411
H	-3.52781978443224	1.90553078613785	-1.93371579416049
C	-2.99394365552036	-0.09766589875229	-4.73510515603607

H	-4.04235334748043	-0.17560190038558	-5.06547807310715
H	-2.50051394208535	-1.05741740201523	-4.94713110180668
H	-2.51763778007728	0.67487650621617	-5.35749499969464
O	0.03697347320023	-1.89744656421125	-0.65385374710659
C	0.87940084663948	-2.86910321616421	-1.05470427438277
C	1.82698982847382	-3.43800740361890	-0.16785388633000
C	1.97117953056405	-2.95493952782109	1.26385351753260
H	1.16611644541841	-2.23866179136070	1.45147304279722
C	3.29676012584673	-2.20806934831665	1.47916584416434
H	4.16176118548221	-2.87592953842842	1.32777408339126
H	3.35340206696110	-1.80717515199614	2.50474536670925
H	3.38196790465912	-1.36469961861781	0.78018745619946
C	1.79533058821158	-4.09217170167952	2.28118866458383
H	2.60515064052146	-4.83740071499792	2.20901369219060
H	0.83718660635502	-4.61400060540215	2.12970699657165
H	1.80469450266216	-3.68962193371757	3.30791706626087
C	2.63646080587426	-4.48330380359303	-0.63351124872892
H	3.37780611247844	-4.91875332567164	0.04147179232822
C	2.50903923718282	-4.98539134375149	-1.92857741647726
H	3.14597579886515	-5.80555451093998	-2.26999912577911
C	1.55286128293736	-4.43115233130451	-2.78254021625473
H	1.44487940229825	-4.82971714691060	-3.79343412845275
C	0.73629855706106	-3.37199848786227	-2.37343706416900
C	-0.34317830941905	-2.82445930011246	-3.29158798373744
H	-0.60679144263962	-1.82046674414320	-2.92960629968452
C	-1.60347551394107	-3.69865639148328	-3.18577054883358
H	-1.37855923536980	-4.73308661791302	-3.49571809571921
H	-2.40639567715493	-3.30998293087102	-3.82902923918298
H	-1.98583411686278	-3.71870093294327	-2.15539805805728
C	0.11063623948685	-2.69517383422386	-4.75057828612012
H	0.30224654482823	-3.67842400088428	-5.20986324769483
H	1.02976718659752	-2.09879511066995	-4.83260888909545
H	-0.66857759181558	-2.20176654288300	-5.35155145307432

Tungsten Selenide

W	0.02099398976632	0.02312517734581	0.02584885975811
Se	0.02373550492542	0.02174777137928	2.35284581945815
O	0.83488290716458	1.68725269124836	-0.60425282816398
C	0.58414128829040	2.88995682952396	-1.14611222482157
C	0.16235500606695	3.96856415019959	-0.33217880693274
C	-0.07046494511020	3.77005322031485	1.15696564304075
H	0.43345846101984	2.83653344767064	1.43838392485008
C	-1.56434760478865	3.58843612672553	1.47485168250172
H	-2.13529935356246	4.49432777231520	1.20868411768392
H	-1.70892148017184	3.39753195989804	2.55102080506305
H	-1.98071976263379	2.73810750390057	0.91837503702771
C	0.53931445918997	4.89230127449552	2.00831387490522
H	1.60406099696851	5.04278184055535	1.77100230880358
H	0.46036590095744	4.64018932069837	3.07857293269257
H	0.01801135891085	5.85200635300331	1.85739757633975
C	-0.02287842469105	5.21929787584333	-0.93437066430917
H	-0.35593127818584	6.06093827836919	-0.32246642787631
C	0.21136586088810	5.41418223465199	-2.29732578220456
H	0.05797497278044	6.39835525955303	-2.74754284082128

C	0.65316439314217	4.34315105720150	-3.07869081688150
H	0.86069851920619	4.50161923568804	-4.13955852841516
C	0.85324994274349	3.07349004495334	-2.52531082243185
C	1.46846586277711	1.95063001414597	-3.34757583000240
H	1.33797489358591	1.01508955811027	-2.78698208451053
C	2.98091479733380	2.19342002941214	-3.48474089358709
H	3.45952383477456	1.37562964507665	-4.04289957139432
H	3.46251582327200	2.24725791332123	-2.49817508306550
H	3.17315849454776	3.13864546872284	-4.01956987628468
C	0.81985405378919	1.75982714364523	-4.72298614322443
H	0.92760238069889	2.65544676325648	-5.35635232698104
H	-0.24928466816994	1.53016883450012	-4.63423594074883
H	1.29971834155302	0.92169948192700	-5.25191572003743
O	-1.73133337186957	0.80840163244932	-0.49333099928370
C	-2.96550667376532	0.42959916346995	-0.88663391464976
C	-3.77979227145515	-0.44221871817472	-0.11802718664192
C	-3.32454612054101	-1.03126446218550	1.20253405765599
H	-2.25344267666854	-0.85180676212703	1.29873460797248
C	-3.51855962923430	-2.55114534639312	1.27726301961523
H	-3.01786167034379	-3.05042485426210	0.43496727538425
H	-4.58345942072349	-2.83647587209031	1.26623300623400
H	-3.08060006041449	-2.94343361125489	2.20917558795640
C	-3.99107161935897	-0.31244294219653	2.38541169293548
H	-3.79354718187156	0.77064159130407	2.34638524237408
H	-3.59551458254420	-0.69433515339547	3.34183508632003
H	-5.08464708967637	-0.45997024325827	2.38433199399999
C	-5.08148479837856	-0.71479926194070	-0.56504546515349
H	-5.71374256666660	-1.37801553794973	0.03019125927896
C	-5.58940746192529	-0.15768015779175	-1.73664461828806
H	-6.60730270813922	-0.38527932584939	-2.06401838273531
C	-4.77924656258374	0.69811231995995	-2.48551538763340
H	-5.17373161689423	1.13784195841251	-3.40326035709963
C	-3.47387176276990	0.99859769866686	-2.08669984865844
C	-2.63117770634892	1.99072676361533	-2.86515240313736
H	-1.57803252301997	1.72483231446714	-2.69896588493165
C	-2.83930972579647	3.40305288603931	-2.29512900468685
H	-2.17701188386299	4.12662145547204	-2.79148346536420
H	-2.61828089181699	3.43113993330207	-1.22133434737352
H	-3.88478827151731	3.72285206679742	-2.44441651578458
C	-2.89332375613285	1.98078404307909	-4.37488270280694
H	-2.19816379866503	2.66816722356368	-4.88099084671539
H	-3.91402980218050	2.31869285979847	-4.61589159900653
H	-2.75532344742160	0.97697874432020	-4.80549850036271
O	-0.79446629544052	-1.64042279154826	-0.60352498965457
C	-0.54374643654648	-2.84191959456399	-1.14816619732297
C	-0.11687783898711	-3.92110912403717	-0.33767827653766
C	0.12151339423134	-3.72480229573926	1.15086523575788
H	-0.38039268754201	-2.79115576922821	1.43536825627408
C	1.61671615088802	-3.54532878384809	1.46362260389581
H	2.18560148965523	-4.45185951389427	1.19521032015885
H	1.76533842826897	-3.35490400548009	2.53932701355781
H	2.03215479861581	-2.69543645213291	0.90576614139303
C	-0.48674457166880	-4.84754528589636	2.00270542373707
H	-1.55256511073007	-4.99602021009189	1.76899675002643

H	-0.40350544523825	-4.59730555376771	3.07308950055444
H	0.03261397873757	-5.80774639876513	1.84827905395171
C	0.06796483425481	-5.17055540453333	-0.94264442389862
H	0.40498931499931	-6.01255858037029	-0.33339860506027
C	-0.17135555915239	-5.36362081301459	-2.30497441269986
H	-0.01803660025091	-6.34679208614096	-2.75740118453655
C	-0.61787554131785	-4.29201873444643	-3.08285167196347
H	-0.82922800128056	-4.44893802048191	-4.14323442123959
C	-0.81799994866157	-3.02363512778358	-2.52654837697161
C	-1.43743691677172	-1.90031066319176	-3.34505780881687
H	-1.31326621358205	-0.96699859414492	-2.77959343619343
C	-2.94814675987414	-2.15007628861230	-3.48760551295672
H	-3.42907457543829	-1.33219529451867	-4.04366367358078
H	-3.43192445172966	-2.20998357027844	-2.50243058654434
H	-3.13462530592279	-3.09390972632037	-4.02687702719692
C	-0.78520251012320	-1.69868776182506	-4.71718231926024
H	-0.88149021879762	-2.59291384414495	-5.35440320999928
H	0.28129120971238	-1.45930999331141	-4.62284596437582
H	-1.27027640167823	-0.86292916637603	-5.24516176446977
O	1.77166978117538	-0.76214908673673	-0.49835942177999
C	3.00588936765319	-0.38495809798600	-0.89291768219386
C	3.82234662821171	0.48545471377250	-0.12482569069212
C	3.36938942633872	1.07540396132948	1.19612420235245
H	2.29831228041364	0.89682573707488	1.29387856914840
C	3.56477802223396	2.59517058464304	1.26981977355479
H	4.62990083542571	2.87955052512597	1.25642442281660
H	3.12915762729655	2.98823836600209	2.20248836383976
H	3.06272725701398	3.09452097097965	0.42839553805481
C	4.03686240635040	0.35659376532576	2.37845634764731
H	3.83826117273616	-0.72632109812193	2.34024900524108
H	3.64291062943614	0.73935073812703	3.33519630541957
H	5.13056315077116	0.50314098890579	2.37591416458799
C	5.12407354407341	0.75601353367698	-0.57282190216312
H	5.75782487512081	1.41816081454974	0.02202121508590
C	5.63026708202567	0.19821382860117	-1.74487776343013
H	6.64834622869226	0.42403877311552	-2.07289716476350
C	4.81811196805628	-0.65617450812950	-2.49308022944366
H	5.21112808364858	-1.09667176192234	-3.41110322402405
C	3.51244056586917	-0.95443733412801	-2.09344205733198
C	2.66813596835792	-1.94462905080602	-2.87264372899830
H	1.61533160069538	-1.68190542238581	-2.70008730510791
C	2.88219481105101	-3.35950407471810	-2.31157052732473
H	2.22067389836086	-4.08208398329246	-2.81048827315107
H	2.66413253954912	-3.39498297980577	-1.23726999138741
H	3.92814177583051	-3.67554351846499	-2.46562568721271
C	2.92272508675512	-1.92529484781926	-4.38371538962352
H	3.94320103073427	-2.25838276992093	-4.63219150382280
H	2.77916141446379	-0.91922525083052	-4.80709166994412
H	2.22696185857771	-2.61163875117350	-4.89040590022490

S5.3 Model Complex [WE(OPh)$_4$]$^-$ XYZ Coordinates

Tungsten Oxide

W	0.00000320679366	-0.00000270407562	-0.00095944423687
O	-0.00053474916124	0.01082689775091	1.72675055809551
O	1.90160252933493	-0.00295206762001	-0.57023176032002
C	2.84771714868956	0.89911323803984	-0.89297270594439
C	3.35784326687752	1.80946584980750	0.06453687853220
H	2.94455771801301	1.81618193770101	1.07490437817379
C	4.36557783597188	2.70059153543084	-0.33208414141123
H	4.75387239175039	3.41812303042974	0.39758260387814
C	4.88534631925171	2.68735472681076	-1.62660478373904
H	5.67400197686320	3.38760563376904	-1.91212939581233
C	4.39205032382139	1.76281219985227	-2.55142439066726
H	4.80182283204603	1.74271748733338	-3.56615506869876
C	3.37406571124053	0.86753646802360	-2.21041335850051
H	2.97344337333744	0.14527031350586	-2.92414630071328
O	0.00654201636537	1.89798746064805	-0.57628902192601
C	-0.88282364469465	2.84697262581283	-0.92235772245126
C	-1.80662539756203	3.36910729889001	0.01591351249977
H	-1.83331943299422	2.96243626829376	1.02860010064505
C	-2.68299151789692	4.38036252822121	-0.40263519716833
H	-3.41060238500643	4.77870927676356	0.31148457806282
C	-2.64286594351650	4.89187302338079	-1.70038513998958
H	-3.33183777274020	5.68402804134051	-2.00308897332516
C	-1.70578423489889	4.38606842209220	-2.60521638835157
H	-1.66462872097857	4.78919069095092	-3.62197907844789
C	-0.82468811979418	3.36296323189739	-2.24230768362480
H	-0.09265756733377	2.95250417659462	-2.94033809908181
O	-1.90120747492020	-0.00297307766240	-0.57192712785585
C	-2.84775285492057	-0.90837823068929	-0.88395618143946
C	-3.35910670110551	-1.80668128922331	0.08435423102383
H	-2.94657902472542	-1.80123149081921	1.09503804165333
C	-4.36691221103210	-2.70220678611576	-0.30204474834337
H	-4.75601783456576	-3.41051708804829	0.43614806217849
C	-4.88576375909799	-2.70474936589385	-1.59700412559659
H	-5.67451995393697	-3.40812765425555	-1.87443499993845
C	-4.39136995594746	-1.79187640668285	-2.53273451455770
H	-4.80036635549814	-1.78407826543712	-3.54794845418338
C	-3.37325453646300	-0.89288084199830	-2.20203554113468
H	-2.97182536564465	-0.17966290361109	-2.92436216071510
O	-0.00663693662458	-1.90507261498182	-0.55283986752917
C	0.88133794398067	-2.86058763127058	-0.88439116438383
C	1.80342193196978	-3.37110273356374	0.06205759275373
H	1.82988679439139	-2.95033208443216	1.06898439171351
C	2.67859709095262	-4.38948778406668	-0.34141370756656
H	3.40490387817992	-4.77880690860201	0.37898268151767
C	2.63882846362363	-4.91930769060706	-1.63179378620992
H	3.32677795613568	-5.71679315492342	-1.92258276195170
C	1.70332460638239	-4.42485267355033	-2.54447252941289
H	1.66231772939690	-4.84231227264023	-3.55543780132717
C	0.82371931286532	-3.39514764011643	-2.19699122096121
H	0.09304009282503	-2.99353100245351	-2.90153826321069

Tungsten Sulfide

W	-0.00000555092692	-0.00000760464856	-0.00088167187542
S	-0.00106028413577	0.01415487094949	2.18819831324489
O	1.89783308721709	-0.00382099074578	-0.56468562597496
C	2.86912412304413	0.85192111900560	-0.94317859903867
C	3.39804339719600	1.82243626072113	-0.05546078162807
H	2.96926586123220	1.92567533434359	0.94308128224670
C	4.44684242034067	2.64017453452394	-0.50013387711315
H	4.84800012195492	3.39833708457923	0.17974566082754
C	4.99157303189942	2.50283651669123	-1.77559487204302
H	5.81352525952263	3.14579724322879	-2.09905941237694
C	4.47560363071616	1.52840077118162	-2.63275271201101
H	4.90111026360963	1.41038335114341	-3.63426609696173
C	3.41618408464429	0.70232295806267	-2.24507958169934
H	3.00399650711575	-0.06005830271536	-2.90886104172863
O	0.02483515770087	1.88108052551113	-0.59312381158984
C	-0.79632758422398	2.86556020675852	-1.00678318958765
C	-1.73125639889636	3.46634687219815	-0.12756191004589
H	-1.83265316626522	3.08448602527618	0.89003362608003
C	-2.51746576139840	4.52325474855508	-0.60668144784063
H	-3.25084794662580	4.98184708896760	0.06397559670815
C	-2.37904490473136	5.00591652147692	-1.90800228269502
H	-2.99704390534701	5.83580703309596	-2.25872405047343
C	-1.43561530967296	4.41961948227367	-2.75472359514647
H	-1.31715910613290	4.79775576217296	-3.77496907462373
C	-0.64263678405995	3.34799121120831	-2.33196405277746
H	0.09556899872128	2.87930486681598	-2.98541899016275
O	-1.89709691121558	-0.00363550542208	-0.56712771080317
C	-2.86791976381190	-0.86469846759849	-0.93480472937542
C	-3.39787470616288	-1.82294158000738	-0.03442256133535
H	-2.96987451546647	-1.91280615059614	0.96574584794093
C	-4.44700581615813	-2.64587081138648	-0.46860946099395
H	-4.84907428621542	-3.39444890281797	0.22127466931185
C	-4.99089258730896	-2.52556566545388	-1.74613846828192
H	-5.81311549804635	-3.17229738974615	-2.06129495708591
C	-4.47363738819748	-1.56331189995323	-2.61619061183834
H	-4.89835731639232	-1.45868697873507	-3.61952211921856
C	-3.41397602578125	-0.73269324530879	-2.23902374337926
H	-3.00106161273843	0.02044212098302	-2.91283609601223
O	-0.02488903441514	-1.88830701991736	-0.56954952228454
C	0.79591171548645	-2.87886758275052	-0.96895013671413
C	1.72932475932761	-3.46862220623356	-0.08069143915419
H	1.82966168440928	-3.07342504672073	0.93190730270756
C	2.51560183684123	-4.53210113366772	-0.54491190893397
H	3.24795374173449	-4.98219782166931	0.13258464011150
C	2.37851472838052	-5.03173585213257	-1.83994107652654
H	2.99659174970433	-5.86640110221294	-2.17899975277300
C	1.43613104322798	-4.45644075587850	-2.69532974016634
H	1.31854740543711	-4.84809793659564	-3.71056308776930
C	0.64315453198577	-3.37898156567990	-2.28766927086785
H	-0.09414697712286	-2.91870099113007	-2.94806386827136

Tungsten Selenide

W	0.00000297232997	-0.00000611688421	-0.00104164450809
Se	-0.00048899066999	-0.00004070571424	2.32595835422492
O	1.85261346897757	-0.00036414929840	-0.63078008107876
C	2.82278298917437	0.75282264666775	-1.17343098331207
C	3.60602130338415	1.60685954778540	-0.36046227373859
H	3.37345637784650	1.69887205505932	0.70194057897838
C	4.64814337389930	2.32224199737586	-0.96338302492433
H	5.25558666080059	2.99419393040177	-0.34915625868983
C	4.92641947786221	2.19572384542832	-2.32612250629305
H	5.74343926715250	2.76454366696623	-2.77636068948647
C	4.15876825389590	1.32709393404712	-3.10650117261411
H	4.38308921235415	1.21756845029732	-4.17202613049735
C	3.10620192391921	0.58998343552325	-2.55236037913805
H	2.49794617690265	-0.09536687154105	-3.14514201665608
O	-0.06483697249560	1.91844555739721	-0.52274608179111
C	-0.94751034993217	2.85996933893767	-0.91744897465619
C	-2.08867193473959	3.20912690186450	-0.14953073768472
H	-2.30702286907551	2.66218593268808	0.76915121743947
C	-2.90561405675799	4.25790753884624	-0.59808383026402
H	-3.78493984325644	4.52781713735280	-0.00468805695752
C	-2.62824464201434	4.95749997368602	-1.77053520519237
H	-3.27938484518523	5.77170977623309	-2.09725056526529
C	-1.50329373133475	4.60501566435068	-2.51872344839918
H	-1.27418271278550	5.14889857506244	-3.44053745909627
C	-0.65965980905103	3.56512963889748	-2.11838035454952
H	0.22493249875678	3.28596157397091	-2.69432005960809
O	-1.85253645892284	0.00036902409083	-0.63078320970272
C	-2.82141522917984	-0.75369051100156	-1.17463230560801
C	-3.60326544857547	-1.61043397943991	-0.36318101364783
H	-3.37065283319803	-1.70388994855356	0.69908348259341
C	-4.64416662628248	-2.32646942785167	-0.96741025762370
H	-5.25058921535568	-3.00043552401552	-0.35436883320919
C	-4.92250007465524	-2.19815748110631	-2.32996079020352
H	-5.73853765654474	-2.76756430916710	-2.78122331952652
C	-4.15610704556025	-1.32706087630583	-3.10883212949891
H	-4.38046719155040	-1.21615437199076	-4.17420721765306
C	-3.10492147478706	-0.58903757206658	-2.55328292548037
H	-2.49772793933730	0.09833157237980	-3.14481682023144
O	0.06431381362573	-1.91835007170531	-0.52273720985255
C	0.94571750707882	-2.86164539872662	-0.91588431438320
C	2.08626768458923	-3.21137933202824	-0.14711256346627
H	2.30530656819850	-2.66316863087854	0.77065564569857
C	2.90158015151033	-4.26224168736962	-0.59356948887321
H	3.78035004552750	-4.53244239939271	0.00052266347418
C	2.62331235006817	-4.96363929645059	-1.76476813291129
H	3.27313169097566	-5.77957101105451	-2.08981541926717
C	1.49904279074154	-4.61069854310960	-2.51364987769562
H	1.26909274950945	-5.15593705304415	-3.43445649454294
C	0.65711864564620	-3.56853103292820	-2.11554295355318
H	-0.22694000347950	-3.28914541368528	-2.69219473107713

References

[1] Persson, C.; Andersson, C. *Inorg. Chim. Acta* **1993**, *203*, 235–238.

[2] Listemann, M. L.; Dewan, J. C.; Schrock, R. R. *J. Am. Chem. Soc.* **1985**, *107*, 7207–7208.

[3] Listemann, M. L.; Schrock, R. R.; Dewan, J. C.; Kolodziej, R. M. *Inorg. Chem.* **1988**, *27*, 264–271.

[4] Kuhn, N.; Henkel, G.; Schumann, H.; Fröhlich, R. *Z. Naturforsch. B* **1990**, *45*, 1010–1018.

[5] Fulmer, G. R.; Miller, A. J. M.; Sherden, N. H.; Gottlieb, H. E.; Nudelman, A.; Stoltz, B. M.; Bercaw, J. E.; Goldberg, K. I. *Organometallics* **2010**, *29*, 2176–2179.

[6] Lochmann, L.; Pospíšil, J.; Lím, D. *Tetrahedron Lett.* **1966**, *7*, 257–262.

[7] Schatz, P. N.; Mowery, R. L.; Krausz, E. R. *Mol. Phys.* **1978**, *35*, 1537–1557.

[8] Mason, W. R. *A Practical Guide to Magnetic Circular Dichroism Spectroscopy*, 1st ed.; John Wiley & Sons, Inc.: Hoboken, NJ, 2007.

[9] Dahm, K. D.; Dahm, D. J. In *Near-Infrared Spectroscopy: Theory, Spectral Analysis, Instrumentation, and Applications*; Ozaki, Y., Huck, C., Tsuchikawa, S., Engelsen, S. B., Eds.; Springer: Singapore, 2021; pp 37–60.

[10] Piepho, S. B.; Schatz, P. N. *Group Theory in Spectroscopy with Applications to Magnetic Circular Dichroism*; John Wiley & Sons: New York, 1983.

[11] Henning, G. N.; McCaffery, A. J.; Schatz, P. N.; Stephens, P. J. *J. Chem. Phys.* **1968**, *48*, 5656–5661.

[12] Katô, H.; Kimura, T. *Bull. Chem. Soc. Japan* **1974**, *47*, 732–734.

[13] Kazmierczak, N. P.; Xia, K. T.; Sutcliffe, E.; Aalto, J. P.; Hadt, R. G. *J. Am. Chem. Soc.* **2025**, *147*, 2849–2859.

[14] Altmann, S. L.; Herzig, P. *Point-Group Theory Tables*, 2nd ed.; University of Vienna PHAIDRA: Wien, 2011.

[15] Neese, F.; Solomon, E. I. *Inorg. Chem.* **1999**, *38*, 1847–1865.

[16] Oganesyan, V. S.; George, S. J.; Cheesman, M. R.; Thomson, A. J. *J. Chem. Phys.* **1999**, *110*, 762–777.

[17] Oganesyan, V. S.; Thomson, A. J. *J. Chem. Phys.* **2000**, *113*, 5003–5017.

[18] Transue, W. J.; Snyder, R. A.; Caranto, J. D.; Kurtz, D. M. J.; Solomon, E. I. *Inorg. Chem.* **2022**, *61*, 16520–16527.

[19] Stephens, P. J. *Adv. Chem. Phys.* **1976**, *35*, 197–264.

[20] Bayliss, S. L.; Laorenza, D. W.; Mintun, P. J.; Kovos, B. D.; Freedman, D. E.; Awschalom, D. D. *Science* **2020**, *370*, 1309–1312.

[21] Furue, S.; Kohmoto, T.; Kunitomo, M.; Fukuda, Y. *Phys. Lett. A* **2005**, *345*, 415–422.

[22] Sutcliffe, E.; Kazmierczak, N. P.; Hadt, R. G. *Science* **2024**, *386*, 888–892.

[23] Chernick, E. T.; Mi, Q.; Kelley, R. F.; Weiss, E. A.; Jones, B. A.; Marks, T. J.; Ratner, M. A.; Wasielewski, M. R. *J. Am. Chem. Soc.* **2006**, *128*, 4356–4364.

[24] Stein, B. W.; Tichnell, C. R.; Chen, J.; Shultz, D. A.; Kirk, M. L. *J. Am. Chem. Soc.* **2018**, *140*, 2221–2228.

[25] Stoll, S.; Schweiger, A. *J. Magn. Reson.* **2006**, *178*, 42–55.

[26] Millhauser, G. L.; Freed, J. H. *J. Chem. Phys.* **1984**, *81*, 37–48.

[27] Saxena, S.; Freed, J. H. *J. Magn. Reson.* **1997**, *124*, 439–454.

[28] Neese, F. *WIREs Comput. Mol. Sci.* **2018**, *8*, e1327.

[29] Neese, F. *WIREs Comput. Mol. Sci.* **2012**, *2*, 73–78.

[30] Ganyushin, D.; Neese, F. *J. Chem. Phys.* **2006**, *125*, 024103.

[31] Stoychev, G. L.; Auer, A. A.; Neese, F. *J. Chem. Theory Comput.* **2017**, *13*, 554–562.

[32] Kollmar, C.; Sivalingam, K.; Helmich-Paris, B.; Angeli, C.; Neese, F. *J. Comput. Chem.* **2019**, *40*, 1463–1470.

[33] Lang, L.; Neese, F. *J. Chem. Phys.* **2019**, *150*, 104104.

[34] Kollmar, C.; Sivalingam, K.; Guo, Y.; Neese, F. *J. Chem. Phys.* **2021**, *155*, 234104.

[35] Guo, Y.; Sivalingam, K.; Neese, F. *J. Chem. Phys.* **2021**, *154*, 214111.

[36] Neese, F. *J. Comput. Chem.* **2023**, *44*, 381–396.

[37] Ugandi, M.; Roemelt, M. *Int. J. Quantum Chem.* **2023**, *123*, e27045.

[38] Becke, A. D. *J. Chem. Phys.* **1993**, *98*, 5648–5652.

[39] Lee, C.; Yang, W.; Parr, R. G. *Phys. Rev. B* **1988**, *37*, 785–789.

[40] Weigend, F.; Ahlrichs, R. *Phys. Chem. Chem. Phys.* **2005**, *7*, 3297–3305.

[41] Zheng, J.; Xu, X.; Truhlar, D. G. *Theor. Chem. Acc.* **2011**, *128*, 295–305.

[42] Andrae, D.; Häußermann, U.; Dolg, M.; Stoll, H.; Preuß, H. *Theoret. Chim. Acta* **1990**, *77*, 123–141.

[43] Grimme, S.; Antony, J.; Ehrlich, S.; Krieg, H. *J. Chem. Phys.* **2010**, *132*, 154104.

[44] Grimme, S.; Ehrlich, S.; Goerigk, L. *J. Comput. Chem.* **2011**, *32*, 1456–1465.

[45] Garcia-Ratés, M.; Neese, F. *J. Comput. Chem.* **2020**, *41*, 922–939.

[46] Neese, F. *J. Comput. Chem.* **2003**, *24*, 1740–1747.

[47] Neese, F.; Wennmohs, F.; Hansen, A.; Becker, U. *Chem. Phys.* **2009**, *356*, 98–109.

[48] Helmich-Paris, B.; De Souza, B.; Neese, F.; Izsák, R. *J. Chem. Phys.* **2021**, *155*, 104109.

[49] Weigend, F. *Phys. Chem. Chem. Phys.* **2006**, *8*, 1057–1065.

[50] Pantazis, D. A.; Chen, X.-Y.; Landis, C. R.; Neese, F. *J. Chem. Theory Comput.* **2008**, *4*, 908–919.

[51] Rao, S. V.; Maganas, D.; Sivalingam, K.; Atanasov, M.; Neese, F. *Inorg. Chem.* **2024**, *63*, 24672–24684.

[52] Neese, F.; Solomon, E. I. *Inorg. Chem.* **1998**, *37*, 6568–6582.
